# Identification and Therapeutic Potential of Polymethoxylated Flavones in *Citri Reticulatae Pericarpium* for Alzheimer’s Disease: Targeting Neuroinflammation

**DOI:** 10.3390/molecules30040771

**Published:** 2025-02-07

**Authors:** Xinyu Wang, Zirong Yi, Yiming Zhang, Jing Zhang, Xueyan Li, Dongying Qi, Qianqian Wang, Xiaoyu Chai, Huan Liu, Guopeng Wang, Yanli Pan, Yang Liu, Guohua Yu

**Affiliations:** 1 School of Chinese Materia Medica, Beijing University of Chinese Medicine, Beijing 102488, China; 17303217015@163.com (X.W.); yixune79@163.com (Z.Y.); 15194178536@163.com (X.L.); 20200941407@bucm.edu.cn (D.Q.); 20220935121@bucm.edu.cn (Q.W.); 20220935120@bucm.edu.cn (X.C.); 20240935145@bucm.edu.cn (H.L.); 2 Institute of Information on Traditional Chinese Medicine, China Academy of Chinese Medical Sciences, Beijing 100700, China; 3 Zhongcai Health (Beijing) Biological Technology Development Co., Ltd., Beijing 101500, China; binglelly@163.com; 4 School of Life Sciences, Beijing University of Chinese Medicine, Beijing 102488, China

**Keywords:** *Citri Reticulatae Pericarpium*, polymethoxylated flavones, anti-neuroinflammation, Alzheimer’s disease, sequential metabolism method, network pharmacology

## Abstract

Neuroinflammation is a significant driving force in the pathogenesis and progression of central nervous system (CNS) disorders. Polymethoxylated flavones (PMFs), the key lipid-soluble constituents in *Citri Reticulatae Pericarpium* (CRP), exhibit excellent blood–brain barrier permeability and anti-inflammatory properties, holding therapeutic potential for CNS disorders. However, the specific bioactive components and therapeutic effects of PMFs in treating CNS disorders are not well understood. This study employed a comprehensive sequential metabolism approach to elucidate the dynamic biotransformation of PMFs in vivo and identified seven brain-targeting components. Subsequently, network pharmacology and experimental validation were utilized to explore the potential mechanisms of PMFs. The results suggested that PMFs have potential therapeutic value for Alzheimer’s disease (AD)-like mice, with the inhibition of neuroinflammation likely being a key mechanism of their anti-AD effects. Notably, sinensetin, tangeretin, nobiletin, and 3,5,6,7,8,3′,4′-heptamethoxyflavone were identified as potent neuroinflammatory inhibitors. This research elucidated the chemical and therapeutic foundations of PMFs, indicating their potential as treatments or nutritional supplements for AD prevention and treatment. Moreover, the integrated triad approach of sequential metabolism, network pharmacology, and experimental validation may serve as a promising strategy for screening bioactive compounds in herbs or functional foods, as well as for elucidating their therapeutic mechanisms.

## 1. Introduction

Disorders of the central nervous system (CNS), such as Alzheimer’s disease (AD), Parkinson’s disease (PD), multiple sclerosis (MS), cerebral ischemic diseases, and brain cancer, are major causes of disability and rank as a high risk factor of death worldwide [1,2]. Despite many therapeutic agents being developed, there is still a shortage of effective therapies for these diseases. An increasing number of recent studies indicate that the inflammatory response leads to neuronal death through multiple pathways and serves as a significant driving force in CNS disorders [3]. In particular, for neurodegenerative diseases such as AD, PD, and MS, inflammation within the CNS is considered to be one of the pathogenic mechanisms of these conditions. As the disease progresses, persistent deposition of β-amyloid protein (Aβ) and dopaminergic deficits, microglial cells, and astrocytes become activated in CNS, triggering the production of various neuroinflammatory-related signaling factors, including cytokines, chemokines, and cell adhesion molecules [4,5]. With the amplification of the inflammatory cascade, brain homeostasis and the blood–brain barrier (BBB) are disrupted, further exacerbating disease progression [6,7]. Therefore, alleviating neuroinflammation is one of the important therapeutic strategies for ameliorating neurological diseases.

Herbs and plant-derived natural compounds have been crucial sources for drug discovery and development. They are characterized by their multi-pathway and multi-target therapeutic effects, as well as having high safety profiles suitable for long-term consumption [8]. *Citri Reticulatae Pericarpium* (CRP), the dried and aged fruit peel of *Citrus reticulata* Blanco and its cultivated forms, exhibits rich pharmacological properties, such as oxidative stress resistance, inflammation inhibition, anti-asthmatic effects, and neuroprotection [9]. For centuries, it has been widely used as a condiment, herbal tea, and therapeutic agent in Eastern and Southeastern Asia. As a condiment, CRP is often braised or fried at high temperatures. As an herbal tea, it is brewed in hot water. As a therapeutic agent, CRP is usually taken after decoction. These processing methods optimize the release of liposoluble components, promoting their absorption by the body. Polymethoxylated flavones (PMFs), the key liposoluble constituents in CRP, exhibit diverse biological activities (Supplementary Table S1). Notably, their excellent BBB permeability and potent anti-inflammatory properties [10,11,12,13] suggest potential therapeutic benefits for the management of CNS disorders. In particular, as part of the daily diet, CRP is regularly consumed through tea and culinary practices in many regions, leading to daily and prolonged intake of PMFs. This consistent intake may confer potential benefits for the prevention, early intervention, and ongoing management of CNS diseases. However, the ameliorative effects of PMFs from CRP on CNS diseases, along with their pharmacological constituents, have yet to be thoroughly investigated.

PMFs in CRP exhibit multi-component characteristics, leading to complex pharmacokinetic and pharmacodynamic profiles [14]. Consequently, it is crucial to investigate the in vivo chemical and metabolic profiles of PMFs. Additionally, assessing the BBB permeability of candidate agents is an important criterion in the development of CNS medications or nutrients. However, this criterion is often overlooked in many studies [15,16]. Previous studies have not provided a complete understanding of the dynamic biotransformation process of PMFs in vivo, and the BBB permeability has been underexamined [17,18]. In this study, we employed a comprehensive sequential metabolism approach utilizing intestinal perfusion with venous sampling (IPVS) [19], an in situ closed-loop technique [20], and UPLC-Q Exactive-Orbitrap HRMS technology as its core analytical platform. This method simulated the dynamic transformation of orally administered multi-component substances within the body, detailing the process of their absorption and metabolism through the gastrointestinal tract and liver, followed by entry into the systemic circulation and further transport to the diseased site. This integrated strategy delineated the dynamic biotransformation process of PMFs and precisely screened the potential pharmacological ingredients for treating CNS disorders.

Network pharmacology leverages leveraging high-throughput omics data analysis, computer simulation, and network database search methods to reveal the network relationships of drug–gene and drug–target interactions with disease. Network pharmacology is characterized by its holistic, systematic, and comprehensive nature, which highly aligns with the multi-component, multi-target, and holistic features of Traditional Chinese Medicine. Currently, network pharmacology has been widely applied in the prediction of the molecular mechanisms, therapeutic efficacy, and adverse reactions of Chinese herbal medicines [21,22]. During this study, network pharmacology was employed to predict the pharmacological and molecular mechanisms of PMFs for treating CNS diseases.

**Figure 1:**
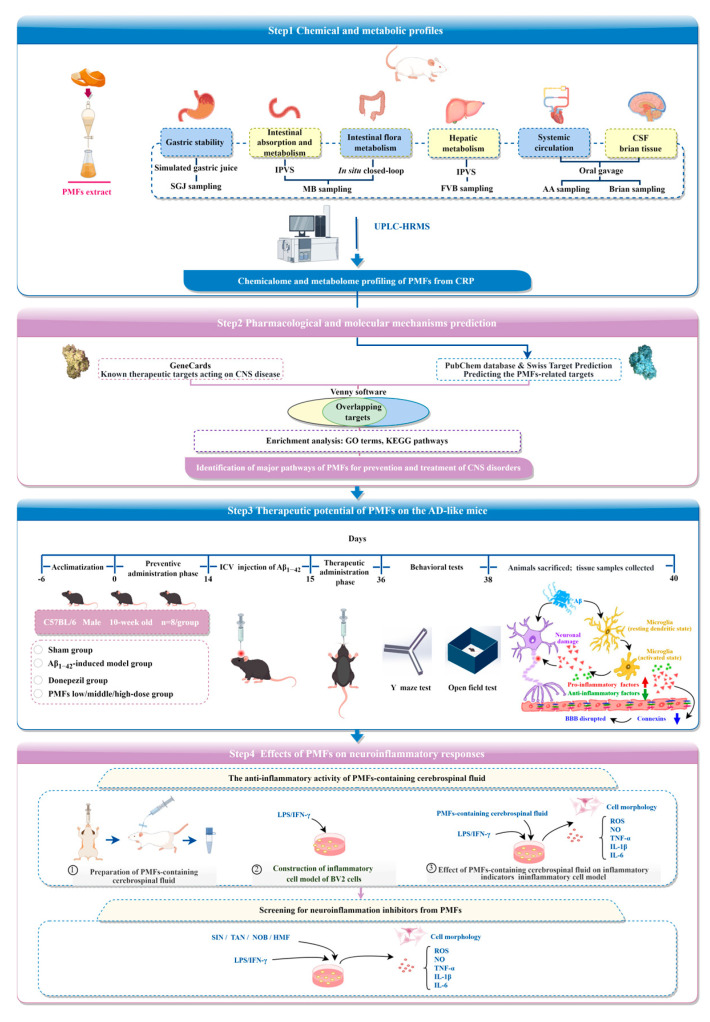
Flowchart of the study design.

In summary, we established a comprehensive sequential metabolism approach to elucidate the dynamic biotransformation of PMFs in CRP and to further analyze the components that enter the brain. Subsequently, network pharmacology was employed to predict the potential pharmacological and molecular mechanisms of PMFs, which were then validated in animal and cell models. Finally, an in vitro screening assay was conducted to screen neuroinflammatory inhibitors from PMFs. This study was designed to provide a detailed chemical and metabolic profile, elucidating the potential bioactive ingredients of PMFs for the amelioration of CNS diseases. Additionally, it sought to delineate the role of PMFs in mitigating CNS disorders via anti-inflammatory pathways and to pinpoint potential neuroinflammatory inhibitors, providing a preliminary research foundation for the development of PMFs as therapeutic agents or dietary supplements. Furthermore, this integrated strategy could effectively be extended to elucidate the efficacy of herbs or functional foods and to screen for active components that ameliorate CNS disorders. The flowchart of our study is illustrated in Figure 1.

## 2. Results

### 2.1. Chemical and Metabolic Profiles of PMFs in CRP

A comprehensive sequential metabolism method was utilized to mimic the biotransformation in vivo of multiple components following oral administration in this research. The components in samples were detected and identified using UPLC-HRMS, including the PMFs extract, the incubation solution of PMFs with simulated gastric juice, plasma samples from the mesenteric vein (for intestinal wall/flora metabolism), femoral vein (for hepatic metabolism), and abdominal aorta, as well as cerebrospinal fluid (CSF) and brain tissue samples. A total of 18 prototype components were identified in the extract of PMFs from CRP (Table 1, and Supplementary Figure S1A) [23,24,25,26]. Nobiletin (NOB, peak 9) was selected as the representative component to elucidate the characteristic fragmentation patterns of PMFs (Supplementary Figure S2). Furthermore, based on the identification results of the PMFs extract, the components of various biological samples were further analyzed. All 18 components were detected in simulated gastric juice (SGJ) group and the mesenteric blood (MB) from intestinal wall/flora metabolism groups. The femoral vein blood (FVB) group yielded seventeen components, the abdominal aorta (AA) group had fifteen, the brain tissue (BT) group contained fourteen, and the CSF group showed seven (Table 1, Supplementary Figure S1B–H). To deeply investigate the biotransformation process of PMFs in vivo, we used NOB as a model to conduct the metabolite analysis and deduce the metabolic pathways [27,28]. As shown in Table 2, a total of 12 metabolites were identified: seven components in the MB from intestinal wall metabolism group, five in the MB from intestinal flora metabolism group, six in the FVB group, three in the AA group, and two in the BT group.

Overall, this study provided a detailed chemical and metabolic profiles, elucidating the potential bioactive ingredients of PMFs for the amelioration of CNS diseases.

### 2.2. Network Pharmacology Analysis

**Figure 2:**
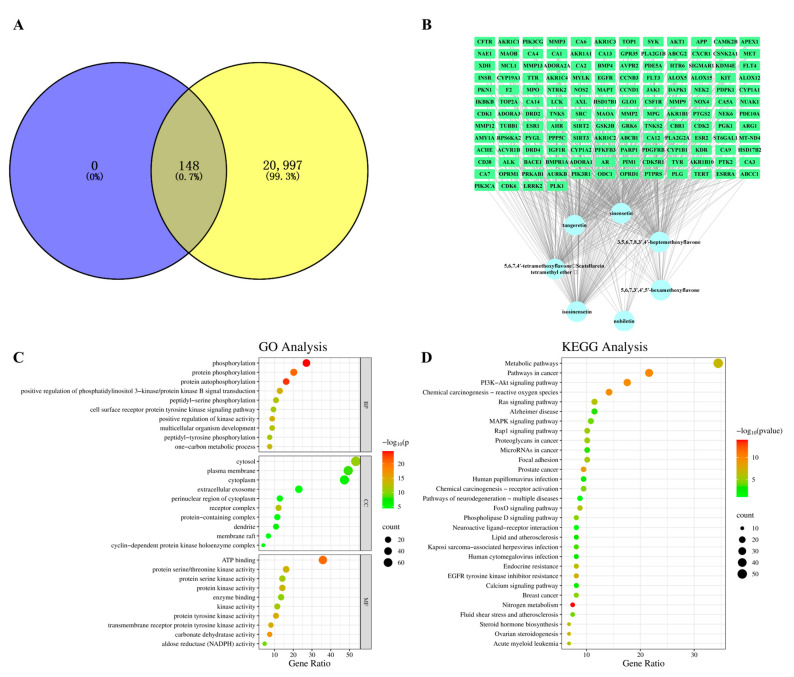
Predicted pathways of ploymethoxylated flavones (PMFs) in treating central nervous system (CNS) disorders. (**A**) Venn diagram of overlapping targets. (**B**) Interaction network of “Components-targets” interaction network. (**C**) GO enrichment analysis (BP, CC, MF). (**D**) KEGG enrichment analysis.

**Figure 3:**
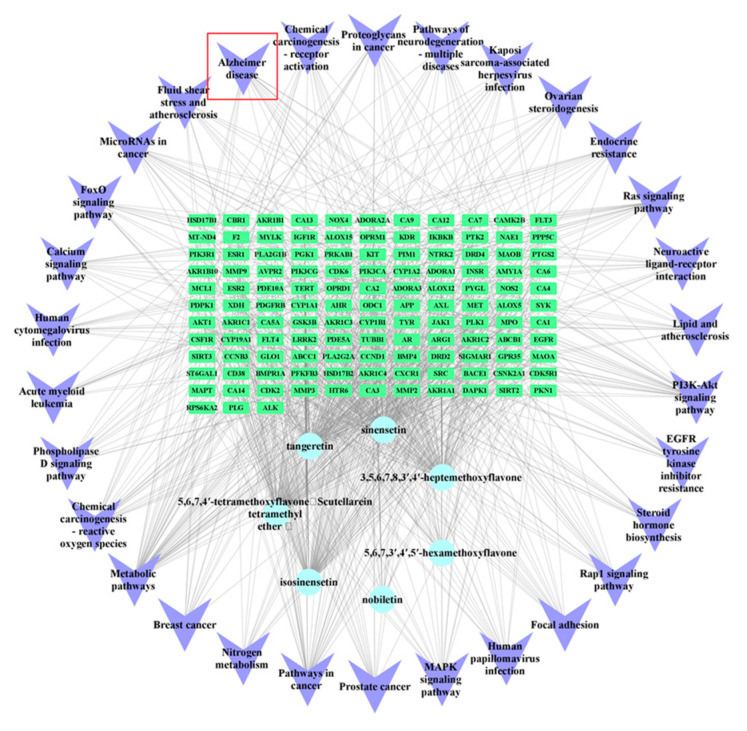
PMFs-targets–pathways interaction network. Blue circles represent PMFs identified in both brain tissue and cerebrospinal fluid (CSF). Green rectangles denote major overlapping targets. Purple arrowhead shapes indicate the top 30 enriched pathways. The red square represents one of the enriched pathways: Alzheimer’s disease.

To conduct precise research on the relationship between PMFs and CNS disorders, seven PMFs identified in both brain tissue and CSF were used for network pharmacology analysis (Table 3). After removing repeated targets, 148 active targets of PMFs and 20,997 CNS disorder-related targets were collected. After processing by Venn diagram analysis, 148 targets were found to be common to both PMFs and CNS disease (Figure 2A). Cytoscape 3.8.2 software was used to construct a “components-targets” interaction network for describing the correspondence between PMFs and disease targets (Figure 2B). Based on the overlapping targets, Gene Ontology (GO) enrichment analysis was utilized to annotate the functions. The findings showed that numerous GO terms were enriched, and Figure 2C shows the top 10 significant terms in the biological processes (BP), molecular functions (MF), and cellular components (CC) categories. To delve deeper into the importance of the key targets, a Kyoto Encyclopedia of Genes and Genomes (KEGG) pathway enrichment analysis was performed. Figure 2D illustrates the top 30 pathways that were enriched by the KEGG pathway enrichment analysis. Based on the pathway enrichment analysis results, a network was constructed that included brain-targeting components, key targets, and significant pathways, with the objective of gaining a comprehensive understanding of the action mechanisms (Figure 3). The enriched pathways suggested that PMFs may intervene in CNS disorders through multiple pathways, including metabolism (such as metabolic pathways, nitrogen metabolism, steroid hormone biosynthesis, ovarian steroidogenesis), infection and immunity (such as human papillomavirus infection, Kaposi sarcoma-associated herpesvirus infection, human cytomegalovirus infection), and signal transduction (PI3K-Akt, MAPK, Rap1 signaling pathway, FoxO signaling pathway, phospholipase D signaling pathway, calcium signaling pathway, neuroactive ligand-receptor interaction). Moreover, the results revealed that neurodegenerative diseases and AD were highly enriched in the major KEGG pathways, suggesting that PMFs might possess potential activity for the prevention and treatment of neurodegenerative diseases, particularly for AD.

### 2.3. Effects of PMFs on the AD-like Mice

#### 2.3.1. Behavioral Tests

**Figure 4:**
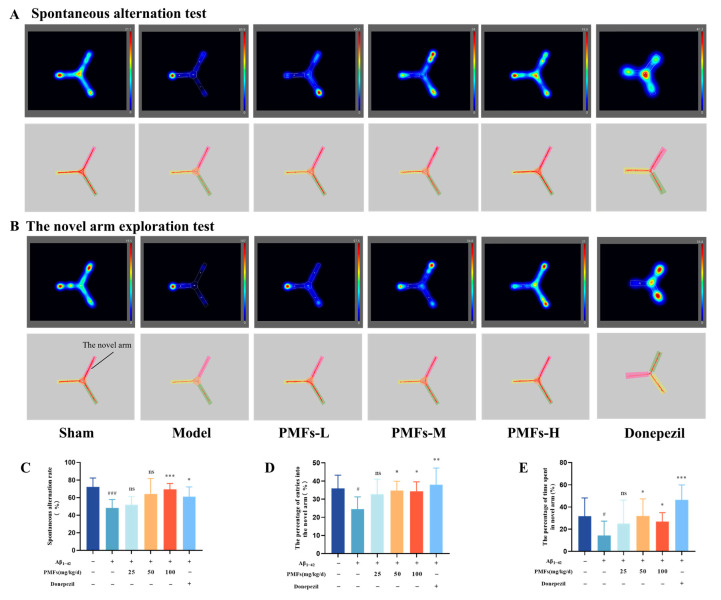
Y-maze test. Representative movement trajectory maps for each group of mice in spontaneous alternation test (**A**) and novel arm exploration test (**B**). (**C**) Spontaneous alternation rate (%). (**D**) Percentage of entries into the novel arm (%). (**E**) Percentage of cumulative time spent in the novel arm (%). ns represents no significant difference. # *p* < 0.05, ### *p* < 0.001, compared with the sham group; * *p* < 0.05, ** *p* < 0.01, *** *p* < 0.001, compared with the model group.

The experimental protocol is detailed in Figure 1. The cognitive function of mice was assessed using the Y-maze spontaneous alternation test and the novel arm exploration test [29,30]. Mice in the sham group displayed robust physical activity, high spontaneous alternation rates, and frequent novel arm entries, indicative of strong spatial memory and exploratory abilities. In contrast, the model group exhibited significantly lower spontaneous alternation rates compared to the sham group (*p* < 0.001, Figure 4A,C). The high-dose PMFs group and the positive drug control group, however, exhibited a significant improvement in spontaneous alternation rates compared to the model group (*p* < 0.001, and *p* < 0.05, respectively). During the novel arm exploration test, the model group exhibited a significant reduction in percentage of entries into the novel arm compared to the sham group (*p* < 0.05, Figure 4B,D). Both the middle- and high-dose PMFs group, as well as the positive drug group, showed a significant increase in the percentage of entries into novel arm compared to the model group (*p* < 0.05, *p* < 0.05, and *p* < 0.01, respectively). Additionally, the model group exhibited a significant decrease in percentage of time spent in the novel arm (*p* < 0.05, Figure 4B,E), i.e., the effect that was reversed by the middle- and high-dose PMFs groups, as well as the positive drug control group (*p* < 0.05, *p* < 0.05, and *p* < 0.001, respectively).

**Figure 5:**
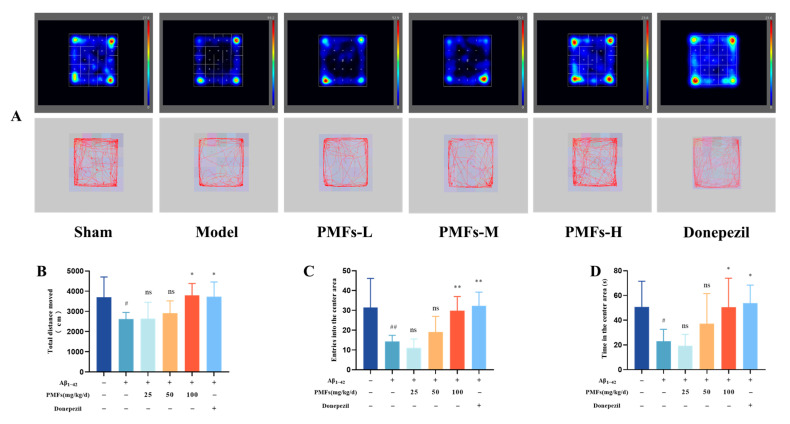
Open field test. (**A**) Representative movement trajectory maps. (**B**) Total distance moved (cm). (**C**) Frequency of entries into the center area. (**D**) Cumulative time exploring in the center area (s). ns represents no significant difference. # *p* < 0.05, ## *p* < 0.01, compared with the sham group; * *p* < 0.05, ** *p* < 0.01, compared with the model group.

The Open Field test was used to assess exploratory, locomotor, and anxiety-like behaviors in mice [31]. Mice in the sham group displayed robust physical activity, frequent entries into the central area, and a prolonged cumulative duration there, indicative of strong exploratory behavior and a healthy mental state. In contrast, the model group showed significant reductions in the total distance moved, the frequency of entries into the central area, and the time spent exploring the central area compared to the sham group (*p* < 0.05, *p* < 0.01, and *p* < 0.05, respectively) (Figure 5). The high-dose PMFs and positive drug groups showed significantly improved behavioral abnormalities in AD-like mice, as Figure 5 shows.

In conclusion, the intervention administration with PMFs and donepezil could ameliorate the abilities of learning, memory, mental state and physical activity of Aβ_1–42_ induced AD-like mice.

#### 2.3.2. Histological Evaluation

**Figure 6:**
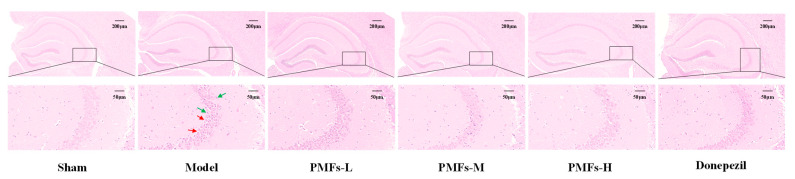
The morphological changes of the neural cells in the hippocampal CA3 region. Representative images of H&E staining of brain tissue from the different treatment groups (scale bar: 200 μm and 50 μm). The green arrows represent nuclear pyknosis, and the red ones represent enlarged neuronal gap.

The accumulation of Aβ leads to neuronal damage or death, ultimately causing cognitive dysfunction [32]. To assess histological alterations in hippocampal neurons, we utilized hematoxylin and eosin (HE) staining. In the sham group, neurons in the hippocampal CA3 region appeared neatly arranged with normal morphology, and no significant nuclear pyknosis was observed (Figure 6). In contrast, the model group displayed pronounced nuclear pyknosis, disarrayed arrangement, and enlarged neuronal gap. Treatment with PMFs and donepezil, however, mitigated these histological abnormalities, restoring the normal structure and arrangement of neurons in the hippocampal CA3 region.

#### 2.3.3. Aβ and P-Tau Measurement

**Figure 7:**
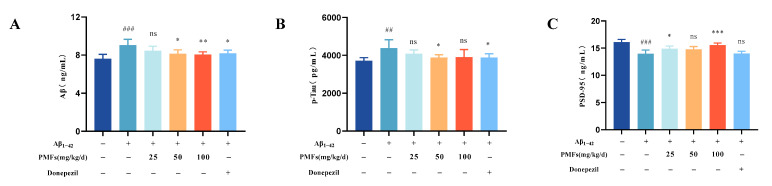
The levels of Aβ, p-Tau, and PSD-95 in the brain tissue from the different treatment groups. (**A**) Aβ, (**B**) p-Tau, (**C**) PSD-95. ns represents no significant difference. ## *p* < 0.01, ### *p* < 0.001, compared with the sham group; * *p* < 0.05, ** *p* < 0.01, *** *p* < 0.001, compared with the model group.

The Aβ deposition and Tau hyper-phosphorylation are the core pathological hallmark of AD [33]. This study evaluated changes in Aβ and p-Tau levels in brain tissue using the ELISA method. The levels of Aβ and p-Tau in the model group were significantly elevated compared to those in the sham group (*p* < 0.001 and *p* < 0.01, respectively) (Figure 7A,B). Both the middle- and high-dose PMFs groups, as well as the positive drug group, exhibited a significant reduction in Aβ levels compared to the model group (*p* < 0.05, *p* < 0.01, and *p* < 0.05, respectively). Similarly, the middle-dose PMFs and positive drug groups significantly decreased p-Tau levels versus the model group (both *p* < 0.05).

#### 2.3.4. Postsynaptic Density Protein 95 (PSD-95) Measurement

Synaptic plasticity impairment and synaptic loss are central events in early cognitive dysfunction, with PSD-95 playing a critical role in synaptic function. In the brains of AD patients, the accumulation of Aβ is closely associated with a reduction in PSD-95 levels [34]. This study evaluated changes in PSD-95 levels in brain tissue using the ELISA method. The level of PSD-95 in the model group were significantly lower than this in the sham group (*p* < 0.001) (Figure 7C). Both the low- and high-dose PMFs groups exhibited a significant increase in PSD-95 levels compared to the model group (*p* < 0.05 and *p* < 0.001, respectively).

#### 2.3.5. Inflammatory Response in Brain Tissue

**Figure 8:**
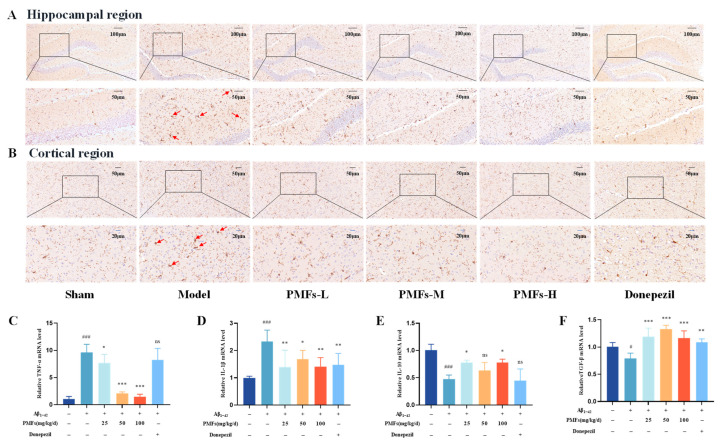
The inflammatory response in the brain. Representative images of immunohistochemical staining for IBA-1 in hippocampal region (scale bar: 100 μm and 50 μm) (**A**) and cortical region (scale bar: 50 μm and 20 μm). The red arrows represent activated microglia with enlarged cell bodies and swollen short branches. (**B**). Expression levels of TNF-α (**C**), IL-1β (**D**), IL-10 (**E**), and TGF-β (**F**) in the brains from the different treatment groups. ns represents no significant difference. # *p* < 0.05, ### *p* < 0.001, compared with the sham group; * *p* < 0.05, ** *p* < 0.01, *** *p* < 0.001, compared with the model group.

Neuroinflammation has been recognized as a pivotal factor in the pathogenesis of AD. Microglia, macrophage-like innate immune cells in the CNS, play a crucial role in neuroinflammation [35]. Immunohistochemistry was employed to assess the quantity and morphology of microglia in the brain. The results illustrated that in the sham group, microglia predominantly displayed a resting morphology with small cell bodies and multiple branches (Figure 8A,B). In contrast, the model group featured activated microglia with enlarged cell bodies and swollen short branches. Compared to the model group, PMF treatment significantly reversed microglial activation in the hippocampal and cortical regions, while donepezil had a less pronounced effect. Additionally, RT-qPCR was employed to assess the expression of inflammatory factors. The results showed that the relative mRNA levels of the pro-inflammatory factors TNF-α and IL-1β in the model group significantly increased compared to those in the sham group (both, *p* < 0.001) (Figure 8C,D). PMFs effectively mitigated these changes, reducing the expression of TNF-α and IL-1β. Meanwhile, the gene expression of anti-inflammatory factors IL-10 and TGF-β in the model group was significantly lower compared to the sham group (*p* < 0.001 and *p* < 0.05, respectively) (Figure 8E,F). Treatment with low- and high-dose PMFs significantly increased IL-10 expression compared to the model group (both, *p* < 0.05), with all PMF doses reversing the decrease in TGF-β expression (both, *p* < 0.001). By contrast, donepezil exhibited a weaker inhibitory effect, only significantly decreasing the expression of IL-1 (*p* < 0.01) and increasing the expression of TGF-β (*p* < 0.01).

In conclusion, PMFs exerted an anti-inflammatory effect in AD-like mice by mitigating microglial activation, downregulating the expression of pro-inflammatory cytokines (TNF-α and IL-1β) and upregulating the expression of anti-inflammatory cytokines (IL-10 and TGF-β).

#### 2.3.6. BBB Functionality Assessment

**Figure 9:**
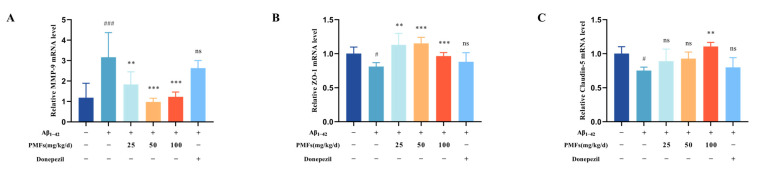
BBB Functionality Assessment. The levels of MMP-9 (**A**), ZO-1 (**B**), and Claudin-5 (**C**) in the brain tissue from the different treatment groups. ns represents no significant difference. # *p* < 0.05, ### *p* < 0.001, compared with the sham group; ** *p* < 0.01, *** *p* < 0.001, compared with the model group.

BBB dysfunction and neuroinflammation interact synergistically, creating a vicious cycle that accelerates the progression of AD [36]. Therefore, we further evaluated the functionality of BBB. RT-qPCR assay showed that the relative mRNA level of matrix metalloproteinase-9 (MMP-9) in the model group was significantly higher than that in the sham group (*p* < 0.001) (Figure 9A). All PMF intervention groups exhibited a significant reduction of MMP-9 levels compared to the model group (*p* < 0.01, *p* < 0.001, and *p* < 0.001, respectively). Furthermore, we examined the levels of tight junction protein closely related to BBB integrity. The results showed that the levels of Zonula Occludens-1 (ZO-1) and Claudin-5 in the model group were significantly reduced compared to those in the sham group (both, *p* < 0.05) (Figure 9B,C). All groups treated with PMFs showed a significant elevation in ZO-1 levels compared to the model group (*p* < 0.01, *p* < 0.001, and *p* < 0.001, respectively). Moreover, the high-dose PMF intervention group significantly reversed the decrease in Claudin-5 levels observed in the model group (*p* < 0.001).

In conclusion, PMFs might preserve BBB integrity in AD-like mice by reducing the expression of MMP-9 and increasing the expression of tight junction proteins (ZO-1 and Claudin-5).

### 2.4. Anti-Inflammatory Effect of PMF-Containing CSF

The CSF containing PMFs was prepared as described in Section 4.4.2. To evaluate the potential cytotoxicity of samples, we investigated the effects of different concentrations of PMF-containing or blank CSF on cells viability. As Supplementary Figure S3A,B shows, PMFs-containing or blank CSF at concentrations ranging from 10% to 50% did not cause significant cytotoxicity. However, cell viability marginally decreased at 40% and 50% blank CSF. Therefore, to ensure the accuracy of the results, concentrations of 10%, 20%, and 30% were ultimately chosen for subsequent experiments.

**Figure 10:**
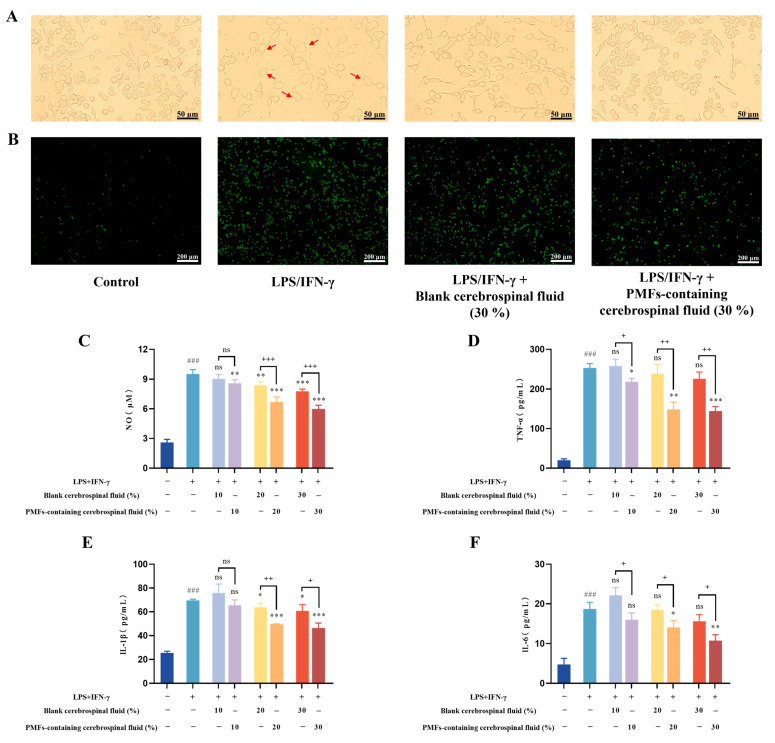
Effects of PMF-containing cerebrospinal fluid on the inflammation in LPS/IFN-γ-induced BV2 cells. (**A**) Morphology of BV2 cells under various treatment condition (scale bar: 50 μm). The red arrows represent activated BV2 microglia cells. (**B**) Intracellular ROS levels in different treatment groups (scale bar: 200 μm). The levels of NO (**C**), TNF-α (**D**), IL-l β (**E**), and IL-6 (**F**) in the different treatment groups. ns represents no significant difference. ### *p* < 0.001, compared with the control group; * *p* < 0.05, ** *p* < 0.01, *** *p* < 0.001, compared with the LPS/IFN-γ-treated group; + *p* < 0.05, ++ *p* < 0.01, +++ *p* < 0.001, compared with the LPS/IFN-γ+blank cerebrospinal fluid treated group.

Subsequently, we investigated the effect of PMF-containing CSF on the LPS/IFN-γ-induced inflammation in BV2 cells. First, the cell morphology was observed under a microscope. As Figure 10A shows, BV2 cells in the control group, under resting conditions, exhibited small cell bodies with a round shape (suspended state) or an epithelial-like morphology with 2–3 pseudopodia (adherent state). In contrast, after stimulation with LPS/IFN-γ, the cells transitioned from a resting to an activated state, characterized by an enlarged cell body, an increase in the proportion of adherent cells, a decrease in pseudopodia or the formation of filopodia, and the manifestation of an amoeboid morphology. However, PMF-containing CSF effectively mitigated these changes, reducing the proportion of activated cells. Subsequently, we measured intracellular ROS levels, which were significantly increased by LPS/IFN-γ stimulation (Figure 10B). The increase in ROS levels was attenuated by the PMF-containing CSF, restoring them to normal levels. Furthermore, the assay results indicated that the NO content was significantly higher in the LPS/IFN-γ group compared to the control group (*p* < 0.001) (Figure 10C). Treatment with 20% and 30% PMF-containing CSF significantly reduced NO levels compared to both the LPS/IFN-γ group and the corresponding blank CSF group (*p* < 0.001). Notably, 20% and 30% blank CSF also demonstrated an inhibitory effect on NO production induced by LPS/IFN-γ (*p* < 0.01). In addition, the levels of pro-inflammatory cytokines TNF-α, IL-1β, and IL-6 were measured to evaluate the anti-inflammatory effects. The ELISA results showed that the content of TNF-α, IL-1β, and IL-6 in BV2 cells were significantly increased in response to LPS/IFN-γ stimulation (*p* < 0.001). Compared to the LPS/IFN-γ group and the corresponding blank CSF group, treatment with 20% and 30% PMF-containing CSF significantly reversed the increased levels of TNF-α, IL-1β, and IL-6 (Figure 10D–F). As with NO, the groups treated with 20% and 30% blank CSF also significantly attenuated the LPS/IFN-γ-induced increase in IL-1β production (*p* < 0.05). Overall, the detection results indicated that PMFs effectively mitigate the inflammatory response induced by LPS/IFN-γ in BV2 microglial cells. Notably, the blank CSF also demonstrated an anti-inflammatory trend, in line with the findings that healthy CSF exerted a protective effect on the brain [37].

### 2.5. Screening for Neuroinflammation Inhibitors from PMFs

**Figure 11:**
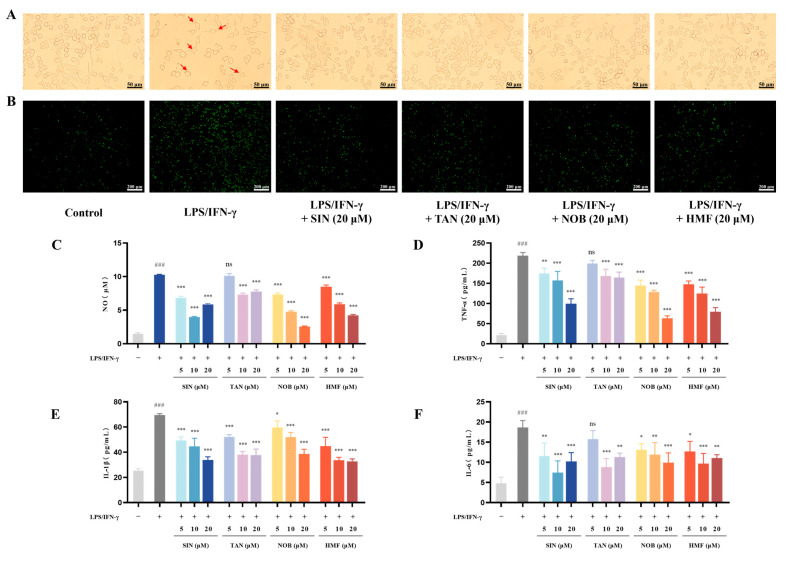
Effects of four monomers on the inflammatory response in LPS/IFN-γ-induced BV2 cells. (**A**) Morphology of BV2 cells under various treatment condition (scale bar: 50 μm). The red arrows represent activated BV2 microglia cells. (**B**) Intracellular ROS levels in different treatment groups (scale bar: 200 μm). The levels of NO (**C**), TNF-α (**D**), IL-l β (**E**), and IL-6 (**F**) in the different treatment groups. ns represents no significant difference. ### *p* < 0.001, compared with the control group. * *p* < 0.05, ** *p* < 0.01, *** *p* < 0.001, compared with the LPS/IFN-γ-treated group.

To screen for potent bioactive monomers, we further investigated the efficacy of four monomers that were identified in both brain tissue and CSF: sinensetin (SIN), tangeretin (TAN), nobiletin (NOB), and 3,5,6,7,8,3′,4′-heptamethoxyflavone (HMF). First, we tested the effects of different concentrations of monomers on cell viability. The results indicated that, at concentrations of 20 μM or lower, all monomers had no significant effect on the viability of BV2 cells. Consequently, concentrations of 5, 10, and 20 μM were chosen for further experimental investigations. Subsequently, the cells were observed under an optical microscope to assess the changes in morphology after treatment with respective groups. Four monomers effectively inhibited the activation of microglia induced by LPS/IFN-γ (Figure 11A). We proceeded to examine their potential capability for scavenging ROS. Four monomers reduced the levels of intracellular ROS compared to the LPS/IFN-γ group (Figure 11B). Moreover, the indicators of inflammation, including NO, TNF-α, IL-1β, and IL-6, were also investigated. Compared with the LPS/IFN-γ group, four monomers reduced the content of NO, and the inhibitory effect was dose-dependent in the group of NOB and HMF (Figure 11C). By contrast, TAN exhibited a weaker inhibitory effect, displaying no significant impact at a concentration of 5 μM. Additionally, four monomers inhibited the production of TNF-α in a dose-dependent manner (Figure 11D). Similarly, four monomers caused the inhibition of the LPS/IFN-γ-induced increase in IL-1β and IL-6 production (Figure 11E,F). In conclusion, both SIN, TAN, NOB, and HMF showed potential anti-neuroinflammatory activity.

## 3. Discussion

Neuroinflammation has been recognized as a significant factor contributing to CNS diseases, exacerbating multiple pathological processes through inflammatory cascades, such as deposition of Aβ, dopaminergic deficits, disruption of brain microenvironment homeostasis, and BBB leakiness [4,5]. PMFs, the key active components in CRP, possess excellent anti-inflammatory activity and superior BBB permeability [10,11,12,13], showing potential for the treatment of CNS disorders. However, the active components and therapeutic effects of PMFs in treating CNS diseases remain unclear.

In this study, a comprehensive sequential metabolism approach was utilized to identify the active components of PMFs. Unlike traditional studies of metabolic characteristics that focus only on isolated fragments of the overall metabolic process [17,18], we developed a holistic, sequential metabolites approach. This method detailed the continuous biotransformation of PMFs from oral administration through major metabolic sites, systemic circulation, and into the brain. The results indicated that 15 of the 18 prototype components in PMFs extract successfully traversed the stomach, intestinal wall, intestinal flora, and liver, reaching systemic circulation. This suggested that PMFs possess chemically stable properties, allowing most components to enter the body in their original forms. Furthermore, among the fifteen prototypical components that entered systemic circulation, fourteen permeated brain tissue and seven entered the CSF, demonstrating their potent ability to permeate the BBB. Additionally, the analysis results of NOB metabolites indicated that the intestinal wall, intestinal flora, and the liver are the primary metabolic sites for PMFs. The structural modifications of the metabolites indicated that the predominant metabolic pathways for PMFs involved demethylation, glucuronidation, hydroxylation, and sulfation [27,28]. Only the metabolites that underwent demethylation and hydroxylation entered the systemic circulation (M7, M8, M10), and among them, M8 and M10 were further transported to the brain tissue. Nevertheless, due to the structural homology of PMFs identified in CRP, a phenomenon of metabolite overlap was observed. Consequently, the detailed characterization of individual PMF metabolites requires further research. In summary, the findings successfully delineated the chemical profile and the map of the dynamic biotransformation of PMFs in CRP and effectively narrowed down the range of potential bioactive components in PMFs for the treatment of brain disorders.

A network pharmacology approach was utilized to investigate the pharmacological and molecular mechanisms underlying the treatment of CNS disorders based on the potential active compounds in PMFs that penetrate the BBB. The results suggested that PMFs might ameliorate CNS diseases through multiple pathways, involving metabolism, infection and immunity, and signal transduction. Key pathways like PI3K-Akt, MAPK, FoxO, and nitrogen metabolism are linked to inflammation. It has been shown that activated microglia in AD could induce PI3K-AKT pathway activation, promoting NF-κB nuclear translocation. As a pivotal transcription factor, NF-κB upregulates the expression of pro-inflammatory mediators, including cytokines (e.g., TNF-α and IL-1β) and enzymes (e.g., COX-1, COX-2, and iNOS) [38]. These mediators amplify neuroinflammation, ultimately contributing to neurodegeneration, neuronal damage, and cognitive decline [39]. Moreover, preclinical evidence suggests that PI3K inhibitors may attenuate neuroinflammatory responses [40]. The mitogen-activated protein kinase (MAPK) signaling pathway plays a pivotal role in modulating inflammatory responses during brain aging, serving as a critical regulator of cellular processes, such as survival, apoptosis, and inflammatory reactions [41]. Key members of the MAPK family, particularly p38 and extracellular signal-regulated kinases 1/2 (ERK1/2), are strongly implicated in the regulation of inflammatory mechanisms [42]. Upon activation, these kinases initiate a phosphorylation cascade that modulates pro-inflammatory transcription factors, thereby triggering and amplifying the inflammatory response cascade [43]. Additionally, the targets of FoxOs include pro-inflammatory molecules like TLR2, TLR4, IL-1β, and TNF-α [44]. Moreover, NO, a key product of nitrogen metabolism, is a pro-inflammatory mediator in AD [45]. Overall, the prediction results suggested that PMFs in CRP could potentially exert anti-CNS disorders effects by inhibiting neuroinflammation. Interestingly, the pathways associated with AD were significantly enriched within the primary KEGG pathways, suggesting that PMFs could be promising candidates for anti-AD treatment. AD, a prevalent neurodegenerative disorder, progressively impairs cognitive function, memory, and reasoning, ultimately resulting in the loss of self-care skills [46]. Current studies indicate that inflammation is a pivotal pathological feature driving the pathogenesis and progression of AD [47]. Inhibiting the inflammatory response may provide a viable approach for the prevention, early intervention, and ongoing management of AD [48]. In subsequent experiments, we investigated the potential of PMFs to ameliorate AD by inhibiting neuroinflammation.

The intracerebroventricular (ICV) injection of Aβ_1–42_ to induce AD-like mice is a commonly used animal model in AD research, exhibiting behavioral and pathological features typical of AD [49,50]. This study revealed that PMFs ameliorated the core symptoms of AD-like mice, including memory decline, anxiety-like states, neural cell damage in hippocampal region, and increased levels of Aβ and p-Tau in the brain. Moreover, the assessment of synaptic function (PSD-95) and BBB-related markers (MMP-9, ZO-1, and Claudin-5) revealed that PMFs exert neuroprotection and improve BBB integrity in AD-like mice. Notably, we focused on the neuroinflammatory inhibitory effects of PMFs, paying particular attention to microglia. As the resident immune cells of the brain, microglia play a pivotal role in regulating inflammatory responses. A moderate activation of microglia and the inflammatory response are essential for neuroprotection. However, persistent and excessive inflammation can lead to a dysregulation of the balance between anti-inflammatory and pro-inflammatory effects, triggering and driving the progression of neurological diseases [4]. The results of animal study revealed that PMFs effectively suppressed microglial activation and regulated the balance of inflammatory factors, thereby alleviating brain inflammation. To further evaluate the anti-neuroinflammatory properties of PMFs, we utilized the LPS/IFN-γ-induced BV2 microglial cell model in subsequent experiments, a widely recognized system for assessing anti-neuroinflammatory efficacy [51]. To ensure experimental rigor, we investigated the anti-inflammatory efficacy of PMFs by analyzing CSF containing PMFs, rather than the extracts alone. Cellular morphology and a series of recognized inflammation-related indicators were utilized to assess the anti-neuroinflammatory potential. We validated the anti-inflammatory efficacy of PMF-containing CSF, indicating that PMFs in CRP not only successfully entered the CSF but also exerted anti-inflammatory effects. In summary, the in vivo and in vitro experimental data supported the therapeutic potential of PMFs for AD, with neuroinflammation inhibition likely playing a pivotal role in their anti-AD mechanism.

Based on the established efficacy of anti-AD and the mechanism of anti-neuroinflammation, further studies were conducted to screen for neuroinflammatory inhibitors among the components of PMFs. We evaluated the anti-inflammatory effects of SIN, TAN, NOB, and HMF, which were simultaneously identified in CSF and brain tissue. The experimental results of the neuroinflammation cell model revealed that SIN, TAN, NOB, and HMF exhibited satisfactory anti-neuroinflammatory properties. NOB inhibits neuroinflammation by preventing NF-κB activation, reducing the expression of NO, IL-6, and TNF-α [52]. TAN exhibits anti-neuroinflammatory activity by suppressing Akt phosphorylation, decreasing mRNA expression of NO, TNF-α, IL-6, and IL-1β [53]. The results of the current study aligned with the previously mentioned findings. By contrast, it is rare for the evaluation of anti-neuroinflammatory properties of SIN and HMF through the microglia to be reported. Consequently, our investigation not only offers critical evidence supporting the anti-neuroinflammatory capabilities of SIN and HMF but also lays a solid groundwork for subsequent inquiries into their potential therapeutic roles in ameliorating CNS disorders.

In summary, these findings provide preliminary evidence supporting the potential of PMFs in CRP as a dietary supplement or therapeutic agent for the prevention and treatment of AD. However, further clinical studies are essential to validate their efficacy and practical application. Additionally, this study employed an integrated approach combining sequential metabolism, bioinformatics, and experimental validation, successfully elucidating the role of PMFs in ameliorating CNS disorders through anti-neuroinflammation, as well as identifying potential active components. Nevertheless, the underlying molecular mechanisms and the in vivo pharmacological efficacy of these active components require further in-depth investigation.

## 4. Materials and Methods

### 4.1. Materials

CRP was procured from Beijing Tong Ren Tang Co., Ltd. (Beijing, China) and was authenticated by Professor Jingjuan Wang at Beijing University of Chinese Medicine (Beijing, China). MS-grade acetonitrile (≥99.9% purity), MS-grade methanol (≥99.9% purity), and formic acid (≥99.9% purity) were obtained from Fisher Scientific (Fairlawn, NJ, USA). Absolute ethanol of high purity (≥99.9%) was supplied by Tianjin Damao Chemical Reagent Factory (Tianjin, China). The compounds NOB (CAS: 478-01-3, ≥98% purity), SIN (CAS: 2306-27-6, ≥98% purity), and TAN (CAS: 481-53-8, ≥98% purity) were acquired from Beijing Beiterenkang Biomedical Technology Co., Ltd. (Beijing, China). HMF (CAS: 1178-24-1, ≥98% purity) was sourced from Nanjing Dilger Medical Technology Co., Ltd. (Nanjing, China). β-Amyloid (1–42) (≥99.66% purity) was acquired from MedChemExpress LLC (Monmouth Junction, NJ, USA). Lipopolysaccharide (Potency ≥ 500,000 EU/mg) was acquired from Beijing Lanboleide Biotechnology Co., Ltd. (Beijing, China). Recombinant Murine IFN-gamma (≥98% purity) was obtained from PeproTech, Inc. (Cranbury, NJ, USA). The IBA-1 antibody (Concentration: 700 μg/mL) was purchased from Proteintech Group, Inc. (Chicago, IL, USA). The horseradish peroxidase (HRP)-labeled goat anti-rabbit (Cat No. 5220-0336) was purchased from SeraCare Life Sciences (Milford, MA, USA). BV2 microglial cells (STR identification. Cat No. CL-0493A) and their corresponding culture medium (Cat No. CM-0493A) were provided by Wuhan Pricella Biotechnology Co., Ltd. (Wuhan, China). A digestive solution (Cat No. C100C1) containing 0.25% pancreatin and 0.01% EDTA, as well as the Cell Counting Kit-8 (Cat No. C6005), were purchased from NCM Biotech Co., Ltd. (Suzhou, China). The NO assay kit (Cat No. S0021S) was procured from Shanghai Biyuntian Biotechnology Co., Ltd. (Shanghai, China). The ELISA Kits for TNF-α (Cat No. SEA133Mu)/IL-1β (Cat No. SEA563Mu)/IL-6 (Cat No. SEA079Mu) were sourced from Wuhan Cloud-Clone Biotechnology Co., Ltd. (Wuhan, China). The ELISA Kits for PSD-95 (Cat No. F9447-B) was sourced from Shanghai Kexing Trading Co., Ltd. (Shanghai, China). The Evo M-MLV RT Mix Kit (Cat No. AG11728), SteadyPure Quick RNA Extraction Kit (Cat No. AG21101), and SYBR Green Premix Pro TaqHS qPCR Kit (Cat No. AG11739) were purchased from Accurate Biotechnology Co., Ltd. (Hunan, China). The hematoxyin and eosin staining solutions (Cat No. B1001) were purchased from BaiQianDu Biotechnology Co., Ltd. (Hunan, China). The NKA-9 adsorption resin (Cat No. 59LSA21LX23) was obtained from Tianjin Berens Biotechnology Co., Ltd. (Tianjin, China). All additional reagents employed were of analytical grade and were readily available commercially.

### 4.2. Preparation of PMFs Extract

The CRP was milled into a powder and approximately 600 g was weighed. Then, the powdered CRP was sonicated twice with 60% ethanol (1:20 solid-to-liquid ratio) at room temperature, with each session lasting 40 min. Following the extraction, the supernatant was combined and concentrated to dryness. This residue was then redissolved in 30% ethanol to prepare a CRP solution with a concentration of 0.5 g/mL. Subsequently, an NKA-9 macroporous resin column chromatography was utilized to enrich PMFs from CRP solution (0.5 g/mL). HPLC with SIN, TAN, NOB, and HMF as standards, was employed to quantify the enriched PMFs extract. Optimal purification: Flow rate of 1 mL/min, sample loading volume of 1.5 g raw drug/g resin, and elution with varying ethanol concentrations. Sequential washing with water was followed by 20% and 40% ethanol to eliminate interfering substances. Ultimately, the column chromatography was rinsed with 95% ethanol to collect the PMFs, which were then concentrated into a dried paste. The PMFs dried paste was accurately weighed and dissolved in a 0.5% sodium carboxymethyl cellulose solution to prepare a 50 mg/mL PMFs solution for animal studies. For the chemical analysis, the PMFs solution was diluted to 5 mg/mL. Four Reference standards were separately dissolved in methanol within 10 mL volumetric flasks and stored at 4 °C. The sample solution was filtered through a 0.22 µm filter before UPLC-HRMS analysis.

### 4.3. Animals

Male Sprague–Dawley rats, weighing 200–250 g, and 10-week-old male C57BL/6J mice, were both obtained from Spfanimals Laboratory Animal Technology Co., Ltd. (Beijing, China). The animals were maintained under controlled environmental conditions with a temperature at 25 ± 2 °C and a relative humidity of 60 ± 10%, with a 12-h light-dark cycle, and were provided with sufficient food and water. They were allowed a 7 day acclimatization period before the commencement of experiments. Furthermore, the rats were subjected to a 12 h fast period prior to the initiation of experimental procedures. All animal handling and experimental protocols were ethically reviewed and approved by the Animal Ethics Committee of Beijing University of Chinese Medicine, with the approval number BUCM-4-2022061502-2062.

### 4.4. Sequential Metabolism of PMFs in CRP

#### 4.4.1. Stability of PMFs Extract in Simulated Gastric Juice

Simulated gastric juice is commonly used to study the stability and biotransformation of chemical compositions under stomach physiological conditions [54]. Simulated gastric juice is typically composed of 0.82 mL of hydrochloric acid and 0.5 g of pepsin (pH 1.5). A total of 1 mL volume of the PMFs extract was added into 50 mL of simulated gastric fluid. The mixture was then incubated at a physiological temperature of 37 °C for a duration of 2 h. The reactions were stopped by adjusting the pH to 6–7 using NaOH (0.1 M) solution to terminate the reaction. The supernatant was filtered through a 0.22 µm filter before UPLC-HRMS analysis.

#### 4.4.2. In Vivo Metabolic Experiments

A comprehensive set of metabolic studies were undertaken in animal models, focusing on the processes of intestinal wall metabolism, intestinal flora metabolism, and hepatic metabolism. The surgical techniques employed for these studies were in accordance with established protocols for IPVS and the in situ closed-loop technique [55,56]. Initially, in preparation for the perfusion surgery, a cohort of 5–7 rats were utilized as blood donors. Whole blood was aspirated from the abdominal aorta and maintained at 37 °C in a water bath. The animal was positioned in a recumbent position on the surgical table after anaesthetization. The supplied blood was infused into the recipient animal via the left external jugular vein using a peristaltic pump to offset surgical blood loss. The abdominal cavity was meticulously opened along the midline to visualize and locate the jejunum and its corresponding mesenteric veins. Subsequently, the PMF solution was infused into the jejunal segment from the proximal end and discharged from the distal end using a syringe pump. Blood was collected from the corresponding location in different experiments. In the intestinal wall metabolism experiment, a catheter was cannulated into the mesenteric vein to collect blood, and the hepatic portal vein was ligatured simultaneously. In the hepatic metabolism experiment, blood was collected through the right external femoral vein. The metabolic transformation of substances within the femoral vein plasma samples was influenced by both the intestines and liver. The in situ closed-loop method was utilized in the intestinal flora metabolism experiment. After the abdominal cavity was opened, the colon segment was located. The PMF solution was injected into the colon, and both ends of the colon were ligated to seal the solution within the segment. The other procedures were the same as those used in the intestinal wall metabolism experiment. During the experiments assessing intestinal wall and hepatic metabolism, the PMF solution was administered at a controlled flow rate of 0.2 mL/min. In all instances, blood was circulated at a consistent flow rate of 0.3 mL/min. The duration for blood sampling typically ranged from 1.5 to 2 h.

Additionally, a separate set of metabolic experiments were carried out with oral drug administration, aiming to identify chemical constituents in the abdominal aorta blood, brain tissue, and CSF. During this process, PMF-containing CSF was collected. The rats were randomly divided into eight groups (*n* = 3). The experimental groups received an oral administration of a PMFs solution (1 g/kg) in a volume of 20 mL/kg, while the control groups were provided with saline. Blood, CSF, and brain tissue samples were collected at designated time points (0.5, 1, 1.5, and 2 h) following intragastric administration. Blood samples collected from the abdominal aorta represent the equilibrium state achieved after metabolic processing by various organs and microbial flora [56]. To ensure uncontaminated brain tissue and CSF free from blood, transcardiac perfusion with saline was conducted before sample collection [57]. After perfusion, the rat was placed in a prone position on the operating table. A needle was carefully inserted at the junction of the atlantooccipital membrane and the dura mater, advancing it until the tip reached the cisterna magna. CSF was then slowly aspirated. Subsequently, the rat was decapitated, and the whole brain was carefully separated from the cranial cavity.

#### 4.4.3. Biological Sample Processing

The plasma sample: The plasma was separated from the whole blood by centrifugating at 4000 rpm for 15 min. Afterward, protein precipitation of the plasma sample was performed using methanol, and the supernatant was obtained by centrifuging at 8000 rpm for 10 min. Then, the supernatant was desiccated under a nitrogen gas stream at a temperature of 40 °C. The resulting residue was subsequently dissolved in 200 µL of methanol for subsequent UPLC-HRMS analysis. The CSF sample: A portion of CSF sample was stored directly at −80 °C for subsequent pharmacological experiments of PMF-containing CSF. The residual CSF was subjected to centrifugation at 12,000× *g* revolutions per min for a duration of 10 min. The resulting supernatant was collected for subsequent UPLC-HRMS analysis. The brain tissue sample: The brain tissue was pulverized in methanol for a duration of 3 min. Subsequently, the supernatant was obtained by centrifuging at 10,000 rpm for a period of 10 min, followed by drying under a nitrogen stream at 40 °C. The residue was dissolved in 200 µL of methanol for subsequent UPLC-HRMS analysis.

### 4.5. UPLC-Q Exactive-Orbitrap HRMS Analysis

The chromatographic separations were conducted by a Waters (Milford, MA, USA) ACQUITY UPLC BEH Shield RP C_18_ column (100 mm × 2.1 mm, 1.7 µm) maintained at a constant temperature of 35 °C. The elution process was executed at a flow rate of 0.3 mL/min, with a sample injection volume of 5 µL. The mobile phase composition involved water acidified with 0.1% formic acid (solvent A) and acetonitrile (solvent B). The gradient elution profile was as follows: 0–10 min, 10–30% B; 10–30 min, 30–95% B; 30–31 min, 95–10% B; 31–35 min, 10% B.

For MS analysis, the conditions were optimized as follows: alternating (−)/(+) ESI modes, with the HESI probe temperature set at 400 °C. The capillary temperature was maintained at 320 °C. The spray voltages were adjusted to +3.5 kV for positive ion mode and −3.0 kV for negative ion mode. The sheath gas (N2) flow rate was set at 35 Arb, while the auxiliary gas flow rate was 10 Arb. Full-scan mass spectra was acquired across a mass range of *m*/*z* 100–1500, with collision energies of 20, 40, and 60 eV for fragmentation. MS/MS experiments were conducted in a data-dependent acquisition mode. Data acquisition and subsequent processing were performed using Xcalibur software (Thermo Fisher Scientific. Released 2017. Xcalibur software, version 4.2. Waltham, MA, USA).

### 4.6. Screening and Identification of Bioactive Compounds in PMFs

Utilizing the Traditional Chinese Medicine System Database and Analysis Platform (TCMSP) (https://old.tcmsp-e.com/tcmsp.php, accessed on 2 November 2024), Herb (http://herb.ac.cn/, accessed on 2 November 2024), the Bio-informatics Analysis Tool for Molecular mechanism of Traditional Chinese Medicine (BATMAN-TCM) (http://bionet.ncpsb.org.cn/batman-tcm/index.php, accessed on 2 November 2024), TCM-Suite (http://tcm-suite.aimicrobiome.cn/, accessed on 2 November 2024) and pertinent literature [23,24,25,26,27,28], an exhaustive search was conducted to identify the potential chemical constituents of CRP using the search terms “chemical name” OR “ingredient” = “*Citri Reticulatae Pericarpium*”. After screening the collected information, detailed specifics about PMFs were obtained. TUPLC-Q Exactive-Orbitrap HRMS was utilized to obtain mass spectrometry data for each sample, combined with the above information to elucidate the chemical constituents.

### 4.7. Network Pharmacology Analysis

Targets Collection: The spatial data file pertaining to brain-targeting PMFs (Table 3) was retrieved from the PubChem database (https://pubchem.ncbi.nlm.nih.gov/, accessed on 18 December 2024). SwissTargetPrediction (http://www.swisstargetprediction.ch, accessed on 18 December 2024) was used to obtain the therapeutic targets. Meanwhile, GeneCards (https://www.genecards.org/, accessed on 18 December 2024) was applied to collect the therapeutic targets of CNS disorders by searching for the keyword “Neurological disorders” and “Central nervous system disorders” and limiting the species with “*Homo sapiens*”.

Network Construction and Analysis: The Venny software 2.1.0 (https://bioinfogp.cnb.csic.es/tools/venny/, accessed on 18 December 2024) was used to identify the overlapping targets between ligands and CNS disorders. Interaction network of “components-targets” was constructed using Cytoscape 3.8.2, a specialized bioinformatics software.

Functional Enrichment Analysis: The intersection genes of components and CNS disorders were uploaded to DAVID data (https://david.ncifcrf.gov/summary.jsp, accessed on 18 December 2024), with “OFFICIAL_GENE_SYMBOL” as the gene identifier and “*Homo sapiens*” as the species. DAVID 6.8 GO was applied to annotate the targets from three aspects: Biological Process (BP), Cellular Component (CC), and Molecular Function (MF). To elucidate the role of target points in signaling pathways, KEGG pathway enrichment analysis was conducted. The top 10 entries (*p* < 0.05) from GO analysis (BP, CC, MF) and the top 30 pathways (*p* < 0.05) from KEGG pathways were selected as the primary gene function enrichment processes and signaling pathways. To predict the mechanisms by which PMFs treat CNS diseases, a network was constructed that included brain-targeting components, key targets, and significant pathways.

### 4.8. Investigation the Effects of PMFs on the AD-like Mice

#### 4.8.1. Groups and Treatment

The intracerebroventricular (ICV) injection technique was used in this study. Aβ_1–42_ was utilized to induce AD-like mice. First, Aβ_1–42_ was processed into its toxic oligomeric form. Briefly, 1 mg of lyophilize peptides was dissolved in 1 mL of hexafluoroisopropanol, mixed by vortexing, and rested at room temperature for 1 h to achieve complete dissolution. After drying at room temperature, the peptide membrane was obtained and resuspended in 20 μL of DMSO. The Aβ_1–42_ oligomers were obtained by diluting a stock Aβ suspension in PBS to a final concentration of 2.5 μg/μL, followed by incubation at 4 °C for 48 h.

A total of 48 mice were used. They were randomly assigned to 6 different groups (*n* = 8). Before the surgery, the mice were given anesthesia and then fixed in a stereotaxic apparatus of brain. At 0.5 mm posterior to the Bregma and 1.0 mm lateral to the sagittal suture, a drill bit was used to carefully drill a hole in the skull. A volume of 1.8 μL of 2.5 μg/μL Aβ_1–42_ solution was slowly injected into bilateral ventricles at an injection depth of 2.0 mm. After the injection was completed, the needle was left in place for 6 min before being slowly withdrawn. The wound was sutured and disinfected with iodophor. The mice were wrapped in cotton to maintain body temperature. The sham group was injected with sterile PBS solution containing 5 % (*v*/*v*) DMSO. To prevent infection, penicillin was administered via intraperitoneal injection for three days postoperatively.

The mice were divided into 6 groups: sham group, Aβ_1–42_-induced model group, donepezil group (1.3 mg/kg/day), low-dose PMFs group (25 mg/kg/day), middle-dose PMFs group (50 mg/kg/day), high-dose PMFs group (100 mg/kg/day). The duration of the study was divided into two phases: a 2 week pre-model preventive administration phase, followed by a 3 week post-model therapeutic administration phase. During the experimental period, the mice in the sham group and Aβ_1–42_-induced model group were administered an equivalent amount of 0.5% sodium carboxymethyl cellulose solution through gavage.

#### 4.8.2. Y Maze Test

The Y maze was used to evaluate the memory and spatial learning of the mice, including the spontaneous alternation test and the novel arm exploration test.

Spontaneous alternation test. To assess spontaneous alternation in the animals, a Y maze of 30 cm × 6 cm × 20 cm was constructed (Y-Mazes, Beijing Zhongshi Dichuang Technology Development Co., Ltd., Beijing, China). One mouse was placed in one arm of the Y maze and allowed to explore freely in the three arms for 5 min [30,31]. An animal’s decision was deemed a correct alternation when it entered three different arms consecutively, whereas any deviation from this pattern was considered an incorrect alternation. The spontaneous alternation among the three arms was recorded and analyzed by an Ethovision XT 15 system (Noldus Ltd., Released 2020. Wageningen, The Netherlands). The percentage alternation was calculated: spontaneous alternation rate (%) = number of spontaneous alternations/(total number of entering the arm − 2) × 100%.

The novel arm exploration test. The test was undertaken in 2 phases: the first phase was the training, during which a partition was employed to obstruct one of the arms, preventing the mice from entering the arm. This arm was designated as the novel arm. Then, the mouse was placed in the start arm and allowed 5 min of exploration in open arms. A total of 4 h after the training, mice performed the second phase. In this phase, the partition was removed, maintaining the novel arm in an open state. The mouse was placed into the start arm of the maze and explored freely in the three arms for 5 min [58]. The movement trajectory of mice in Y maze was recorded and analyzed by an EthoVision XT system. Novel arm exploration was measured by normalizing novel arm exploration to total exploration: (i) The percentage of entries into the novel arm = number of entries into novel arm/number of entries into all arms × 100%. (ii) The percentage of time spent in novel arm = cumulative time in novel arm/cumulative time in all arms × 100%.

#### 4.8.3. Open Field Test

To assess the ability of exploratory, locomotor, and anxiety-like behavior in mice, an open field of 50 cm × 50 cm × 40 cm was constructed (Open Field, Beijing Zhongshi Dichuang Technology Development Co., Ltd., Beijing, China). The mouse was gently placed in the center of the open field and allowed to move freely for 10 min [59]. Total distance moved, the frequency of entries into the center area, and time exploring in the center area were analyzed using EthoVision XT system.

#### 4.8.4. Tissue Preparation

Animals were sacrificed after behavioral tests to collect brain tissue. Firstly, the mice were rendered unconscious through an intraperitoneal injection of anesthetics. Then, the mice were placed in a supine position on the operating table and perfused transcardially with ice-cold saline. After decapitation, brain tissue was removed and weighed. The left brain tissue was stored at −80 °C for biochemical analysis, and the right brain tissue was immersed in 4% paraformaldehyde for HE staining.

#### 4.8.5. HE Staining for Histological Evaluation

HE staining was applied to evaluate the morphological changes in the hippocampal region [60]. The brain tissue was removed from the fixative, then dehydrated using a series of alcohol concentrations, followed by paraffin treatment and embedding. Paraffin-embedded brain tissue was cut into 4 μm sections using a pathological slicer (RM2016, Leica Biosystems, Shanghai, China), and sections were stained using a standard HE staining protocol. HE images of brain tissue sections were obtained using the imaging system (DS-U3, Nanjing Kangni Mechanical&Electrical Co., Ltd., Tokyo, Japan). The histological evaluation was conducted by an individual who was unaware of the experimental groups.

#### 4.8.6. Activated Microglial Evaluation

Immunohistochemistry was applied to evaluate the quantity and morphology of microglia in hippocampal and cortical region [29]. The fixed brain tissues underwent dehydration, embedding, and sectioning before being incubated with the IBA-1 antibody. Then secondary antibodies were incubated. Images of brain tissue sections were obtained using the imaging system (DS-U3, Nanjing Kangni Mechanical&Electrical Co., Ltd., Tokyo, Japan).

### 4.9. Cell Culture and Treatment

BV2 microglial cells were cultured in a 37 °C, 5% CO_2_ cellular incubator. Cells were cultured to approximately 80% confluence, and then passaged for subsequent experiments. After cells reached the logarithmic growth phase, they were stimulated with LPS (1 µg/mL)/IFN-γ (2.5 ng/mL) for 24 h to establish a cell model of analog neuroinflammation. Broadly, the experimental groups included the control group, LPS/IFN-γ alone group, and LPS/IFN-γ combined with different components group. In the LPS/IFN-γ combined with different components (blank CSF, PMFs-containing CSF, PMFs monomers) treated groups, BV2 cells were first incubated with designated component for 2 h. After preincubation, LPS/IFN-γ were added to the culture medium, and the cells were then co-incubated with LPS/IFN-γ in the presence of designated component for an additional 24 h.

### 4.10. Cell Viability Assay

BV2 cells were moved into 96-well plates at a seeding density of 1 × 10^5^ cells/mL, with each well receiving 0.1 mL of cell suspension. Following cell adhesion, the designated component was added to the culture medium, and the cells were then co-incubated with the designated component in the cell culture incubator for 24 h. Subsequently, cell viability was measured according to the Cell Counting Kit-8’s instructions.

### 4.11. NO and ROS Measurement

The supernatant from the cell culture was collected and centrifuged at 3000 rpm for a duration of 5 min to eliminate dead cells and cellular debris. The concentration of NO within the cell culture medium was measured using the Griess method.

The cell culture solution was removed from each well. Subsequently, 1 mL of fresh solution containing a fluorescent probe for ROS was added, and the plates were incubated for an additional 0.5 h in the incubator. The cells were subsequently observed and imaged using a fluorescence microscope (U-HGLGPS, Olympus Corporation, Tokyo, Japan).

### 4.12. ELISA Analyses

Brain samples were ground with PBS solution, then centrifuged at 5000 rpm at 4 °C for 10 min, and the brain extract was collected. The supernatant from the cell culture was collected and centrifuged at 3000 rpm for a duration of 5 min to eliminate dead cells and cellular debris. The concentrations of PSD-95, Aβ, P-Tau, TNF-α, IL-1β, and IL-6 in the cell culture medium or brain extract were quantified using ELISA assays, in accordance with the manufacturer’s protocols.

### 4.13. RT-qPCR Analyses

Tissues were homogenized, and RNA was extracted using the SteadyPure Quick RNA Extraction Kit, followed by reverse transcription to cDNA with the Evo M-MLV RT Mix Kit. The cDNA was then combined with gene-specific primers and the SYBR Green Premix Pro TaqHS qPCR Kit for qPCR reactions. The amplification conditions for qPCR included an initial denaturation at 95 °C for 30 s, followed by 40 cycles of 95 °C for 3 s (denaturation) and 60 °C for 20 s (annealing/extension). Gene expression was normalized to GAPDH and calculated using the 2^−ΔΔCt^ method. All RT-qPCR analyses were performed in triplicate on the FQD 96A RealTime Fluorescence Quantitative PCR System (QuantStudio 7 Flex, Applied Biosystems, Inc., Carlsbad, CA, USA). The primer sequences used in these analyses are provided in Table 4.

### 4.14. Statistical Analysis

Statistical analysis was conducted employing GraphPad Prism software, version 9.5.0. (GraphPad Software, Inc., Released 2023. La Jolla, CA, USA). Data derived from three separate replicate experiments were collectively analyzed, and the findings were presented as the mean ± standard deviation (SD). The data from multiple groups were evaluated through one-way analysis of variance (ANOVA), with subsequent testing via the Sidak multiple comparisons post hoc analysis. Statistical significance was determined at *p* < 0.05.

## 5. Conclusions

This study developed a comprehensive sequential metabolism approach that emulated the process of continuous biotransformation of bioactive constituents after oral administration. This approach innovatively characterized the chemical and metabolic profiles of PMFs in CRP and identified seven brain-targeting components as potential pharmacological ingredients for treating CNS disorders. Following the identification of potential constituents, network pharmacology analysis was utilized to predict the pharmacological and molecular mechanisms. The results indicated that pathways associated with AD and inflammation were highly enriched, suggesting that PMFs may exert beneficial effects in ameliorating AD by regulating the inflammatory response. Subsequent in vivo and in vitro experimental validation suggested that PMFs hold potential therapeutic value for AD, with the inhibition of neuroinflammation likely being a key mechanism of their anti-AD effects. The integration of bioinformatics predictions with experimental validation significantly enhanced the efficiency and accuracy of research in elucidating therapeutic action. Additionally, four monomers, SIN, TAN, NOB, and HMF, were identified as potent inhibitors of neuroinflammation. These findings elucidated the chemical and functional underpinnings of PMFs in CRP, suggesting that PMFs have potential as therapeutic agents or dietary supplements in the amelioration of AD.

## Figures and Tables

**Table 1:** Identification of prototypical compounds from polymethoxylated flavones (PMFs) in *Citri Reticulatae Pericarpium* (CRP) and the absorbed components in different biological samples.

No.	t*_R_* /min	Observed Mass	Error /ppm	MS/MS Fragments	Molecular Formula	Proposed Compounds	PMFs	SGJ	MB-W	MB-F	FVB	AA	CSF	BT	Ref.
1	12.20	[M + H]^+^ 375.1434	−1.065	211.0604, 196.0369, 168.0415, 150.0314	C_20_H_22_O_7_	5,6,7,3′,4′-pentamethoxyflavanone	Y	Y	Y	Y	Y	N	N	N	[23]
2	13.07	[M + H]^+^ 373.1282	0.136	358.1048, 343.0814, 315.0865, 181.0132, 153.0183	C_20_H_20_O_7_	Isosinensetin	Y	Y	Y	Y	Y	Y	Y	Y	[24]
3	13.72	[M + H]^+^ 403.1389	0.106	388.1147, 373.0921, 327.0861	C_21_H_22_O_8_	5,7,8,3′,4′,5′-hexamethoxyflavone	Y	Y	Y	Y	Y	Y	N	Y	[23]
4	13.79	[M + H]^+^ 389.1234	0.658	374.1000, 359.0761, 328.0958, 313.0702	C_20_H_20_O_8_	3′(4′)-monohydroxy-5,6,7,8,4′(5,6,7,8,3′)-pentamethoxyflavone	Y	Y	Y	Y	Y	Y	N	Y	[23]
5	14.17	[M + H]^+^ 373.1281	−0.266	358.1042, 343.0814, 312.0993, 329.1021, 357.0967	C_20_H_20_O_7_	Sinensetin *	Y	Y	Y	Y	Y	Y	Y	Y	*
6	14.30	[M + H]^+^ 375.1441	0.641	211.0604, 196.0369	C_20_H_22_O_7_	5,7,8,3′,4′-Pentametho-xyflavanone	Y	Y	Y	Y	Y	Y	N	Y	[23]
7	14.51	[M + H]^+^ 389.1233	0.427	374.1002, 359.0761, 341.0656, 316.0576	C_20_H_20_O_8_	Monohydroxy-pentamethoxyflavone	Y	Y	Y	Y	Y	Y	N	N	[25]
8	14.78	[M + H]^+^ 403.1387	0.016	388.1135, 373.0919, 327.0866	C_21_H_22_O_8_	5,6,7,3′,4′,5′-hexamethoxyflavone	Y	Y	Y	Y	Y	Y	Y	Y	[23]
9	15.09	[M + H]^+^ 403.1387	−0.074	388.1151, 373.0919, 355.0811, 327.0863	C_21_H_22_O_8_	Nobiletin *	Y	Y	Y	Y	Y	Y	Y	Y	*
10	15.43	[M + H]^+^ 343.1176	−0.014	328.0937, 313.0706, 285.0758, 181.0133, 153.0184, 133.0649	C_19_H_18_O_6_	5,6,7,4′-tetramethoxyflavone	Y	Y	Y	Y	Y	Y	Y	Y	[23]
11	15.67	[M + H]^+^ 433.1494	0.234	418.1245, 403.1024, 388.0774, 360.0845, 385.0923, 345.0604	C_22_H_24_O_9_	3,5,6,7,8,3′,4′-heptemethoxyflavone *	Y	Y	Y	Y	Y	Y	Y	Y	*
12	15.70	[M + H]^+^ 389.1231	−0.036	374.1004, 359.0761, 356.0887, 341.0656, 197.0883, 169.0132	C_20_H_20_O_8_	Monohydroxy-pentamethoxyflavone	Y	Y	Y	Y	Y	Y	N	Y	[25]
13	16.28	[M + H]^+^ 373.1282	0.136	358.1043, 343.0813, 325.0714, 300.0622, 297.0757	C_20_H_20_O_7_	Tangeretin *	Y	Y	Y	Y	Y	Y	Y	Y	*
14	16.38	[M + H]^+^ 419.1330	−1.572	404.1095, 389.0865, 371.0760, 361.0908	C_21_H_22_O_9_	5-monohydroxy-6,7,8,3′,4′,5′-hexamethoxyflavone	Y	Y	Y	Y	N	N	N	N	[23]
15	16.55	[M + H]^+^ 359.1124	−0.277	344.0887, 329.0660, 311.0545, 301.0695, 163.0755, 138.9975	C_19_H_18_O_7_	Monohydroxy-tetramethoxyflavone	Y	Y	Y	Y	Y	Y	N	Y	[25]
16	17.03	[M + H]^+^ 403.1388	0.076	388.1156, 373.0919, 327.0872	C_21_H_22_O_8_	Hexamethoxyflavone	Y	Y	Y	Y	Y	Y	N	Y	[23]
17	17.41	[M + H]^+^ 389.1231	−0.036	374. 1000, 359. 0761, 341. 0655, 331. 0811, 197. 0082	C_20_H_20_O_8_	5-hydroxy-6,7,8,3′,4′-pentamethoxyflavone	Y	Y	Y	Y	Y	Y	N	Y	[26]
18	17.92	[M + H]^+^ 419.1338	0.313	404.1100, 389.0868, 371.0758	C_21_H_22_O_9_	Monohydroxy-hexamethoxyflavone	Y	Y	Y	Y	Y	N	N	N	[25]

Note: t*_R_*: retention time; *: compounds identified by reference standards; Y: Detected; N: Not Detected; PMFs: PMFs Extract; SGJ: simulated gastric juice; MB-W: mesenteric blood from intestinal wall metabolism group; MB-F: mesenteric blood from intestinal flora metabolism group; FVB: femoral venous blood; AA: abdominal aorta; CSF: cerebrospinal fluid; BT: brain tissue.

**Table 2:** Identification of metabolites of nobiletin in different biological samples.

No.	t*_R_* /min	Observed Mass	Error /ppm	Molecular Formula	MS/MS Fragments	SGJ	MB- W	MB- F	FVB	AA	CSF	BT	Metabolites	Structure of Prediction
M1	8.78	[M + H]^+^ 595.1661	0.661	C_27_H_30_O_15_	419.1342, 389.0872, 404.1107, 371.0768, 595.1672, 403.1023, 346.0687, 165.0548	N	Y	N	Y	N	N	N	Hydroxylation + Glucuronidation	C_15_H_12_O_2_+6OCH_3_+OH+GluA-12H
M2	9.44	[M + H]^+^ 595.1663	0.964	C_27_H_30_O_15_	419.1342, 389.0870, 404.1104, 371.0764, 346.0688, 343.0444, 374.0628, 403.1026	N	Y	N	N	N	N	N	Hydroxylation + Glucuronidation	C_15_H_12_O_2_+6OCH_3_+OH+GluA-12H
M3	9.54	[M + H]^+^ 537.1243	0.835	C_24_H_24_O_14_	211.0604, 361.1285, 196.0369, 183.0291, 360.1165, 167.0339, 330.0693, 177.0547	N	N	Y	N	N	N	N	Demethylation + Glucuronidation	C_15_H_10_O_2_+3OCH_3_+3OH+GluA-8H
M4	9.68	[M + H]^+^ 565.1556	0.704	C_26_H_28_O_14_	389.1234, 359.0763, 257.081, 565.1561, 85.0291, 565.372, 390.127	N	N	Y	Y	N	N	N	Demethylation + Glucuronidation	C_15_H_10_O_2_+5OCH_3_+OH+GluA-8H
M5	10.92	[M + H]^+^ 551.1401	1.104	C_25_H_26_O_14_	375.108, 345.0609, 342.0739, 360.0839, 376.1107, 359.0764, 85.0291, 270.0887	N	Y	N	N	N	N	N	Demethylation + Glucuronidation	C_15_H_10_O_2_+4OCH_3_+2OH+GluA-8H
M6	10.98	[M + H]^+^ 565.1559	1.341	C_26_H_28_O_14_	389.1236, 331.0816, 356.0894, 313.0708, 374.0999, 390.12732, 359.0766, 328.0945	N	Y	N	N	N	N	N	Demethylation + Glucuronidation	C_15_H_10_O_2_+5OCH_3_+OH+GluA-8H
M7	12.30	[M + H]^+^ 389.1234	0.658	C_20_H_20_O_8_	389.1237, 359.0766, 169.0134, 331.0817, 341.066, 390.1272, 316.0580, 374.1003	N	Y	Y	Y	Y	N	N	Demethylation + Hydroxylation	C_15_H_10_O_2_+5OCH_3_+OH-6H
M8	15.88	[M + H]^+^ 389.1231	−0.113	C_20_H_20_O_8_	389.1234, 331.0814, 356.0893, 313.0709, 359.0765, 341.0660, 328.0945, 390.1264	N	Y	Y	N	Y	N	Y	Demethylation + Hydroxylation	C_15_H_10_O_2_+5OCH_3_+OH-6H
M9	15.91	[M + H]^+^ 375.1074	0.336	C_19_H_18_O_8_	375.1077, 345.0607, 356.0892, 317.0657, 313.0707, 331.0813, 360.0830, 374.0966	N	N	Y	N	N	N	N	Demethylation	C_15_H_10_O_2_+4OCH_3_+2OH-6H
M10	17.38	[M + H]^+^ 389.1231	−0.113	C_20_H_20_O_8_	389.1235, 359.0764, 341.0661, 197.0083, 169.0134, 163.0756, 390.1267, 316.0581	N	N	N	Y	Y	N	Y	Demethylation + Hydroxylation	C_15_H_10_O_2_+5OCH_3_+OH-6H
M11	17.50	[M − H]^−^ 467.0661	3.943	C_20_H_20_O_11_S	387.1093, 372.0857, 357.0623, 342.0385, 467.0659, 327.0153, 299.0203, 388.1125	N	N	N	Y	N	N	N	Demethylation + Sulfation	C_15_H_10_O_2_+5OCH_3_+OH+SO_3_-6H
M12	20.78	[M − H]^−^ 453.0505	4.065	C_19_H_18_O_11_S	373.0933, 343.0463, 358.0699, 328.023, 374.0968, 453.0513, 300.0279, 359.0729	N	Y	N	Y	N	N	N	Demethylation + Sulfation	C_15_H_10_O_2_+4OCH_3_+2OH+SO_3_-6H

Note: t*_R_*: retention time; Y: Detected; N: Not Detected; SGJ: simulated gastric juice; MB-W: mesenteric blood from intestinal wall metabolism group; MB-F: mesenteric blood from intestinal flora metabolism group; FVB: femoral venous blood; AA: abdominal aorta; CSF: cerebrospinal fluid; BT: brain tissue.

**Table 3:** Chemical structures of PMFs used for network pharmacology analysis.

Molecule Name	Structure
Isosinensetin	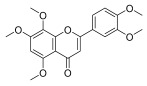
Sinensetin	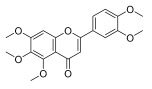
5,6,7,3′,4′,5′-hexamethoxyflavone	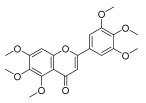
Nobiletin	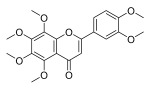
5,6,7,4′-tetramethoxyflavone	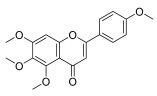
3,5,6,7,8,3′,4′-heptemethoxyflavone	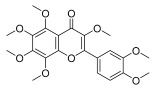
Tangeretin	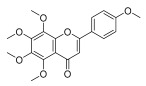

**Table 4:** PCR primers for detecting mRNA expression in this study.

Gene Symbol	Forward Primer	Reverse Primer
TNF-α	CCCTCACACTCACAAACCAC	ACAAGGTACAACCCATCGGC
IL-1β	CACAGCTCTGGAGATGGTGA	CTTTCAAGCTTGGGCACTTC
IL-10	GGTTGCCAAGCCTTATCGGA	GACACCTTGGTCTTGGAGCTTA
TGF-β	CCTCGAGACAGGCCATTTGT	GCCAGCTGACTGCTTTTCTG
MMP-9	TCTAGGCCCAGAGGTAACCC	AGTCGAATCTCCAGACACGC
Claudin-5	GTTAAGGCACGGGTAGCACT	TACTTCTGTGACACCGGCAC
ZO-1	CTCAAGTTCCTGAAGCCCGT	GCAAAAGACCAACCGTCAGG
GAPDH	AGGTCGGTGTGAACGGATTTG	GGGGTCGTTGATGGCAACA

## Data Availability

The original contributions presented in the study are included in the article, and further inquiries can be directed to the corresponding author.

## Supplementary Materials

The following supporting information can be downloaded at: https://www.mdpi.com/article/10.3390/molecules30040771/s1, Figure S1. Total ion chromatogram (TIC); Figure S2. Fragmentation pathways of NOB in positive ion mode; Figure S3. Cell viability test. Table S1. Summary of pharmacokinetic and pharmacodynamic properties of PMFs. References [61,62,63,64,65,66,67,68,69,70,71,72,73,74,75,76,77,78,79,80,81,82,83,84,85] are cited in the Supplementary Materials.
