# Supplementary material for: Identification and Therapeutic Potential of Polymethoxylated Flavones in Citri Reticulatae Pericarpium for Alzheimer’s Disease: Targeting Neuroinflammation
Source: Molecules. 2025 Feb 7;30(4):771. doi: 10.3390/molecules30040771 (PMC11857992; doi:10.3390/molecules30040771)
Supplement: Supplementary file 1 [file molecules-30-00771-s001.zip › molecules-3441971-supplementary.pdf]

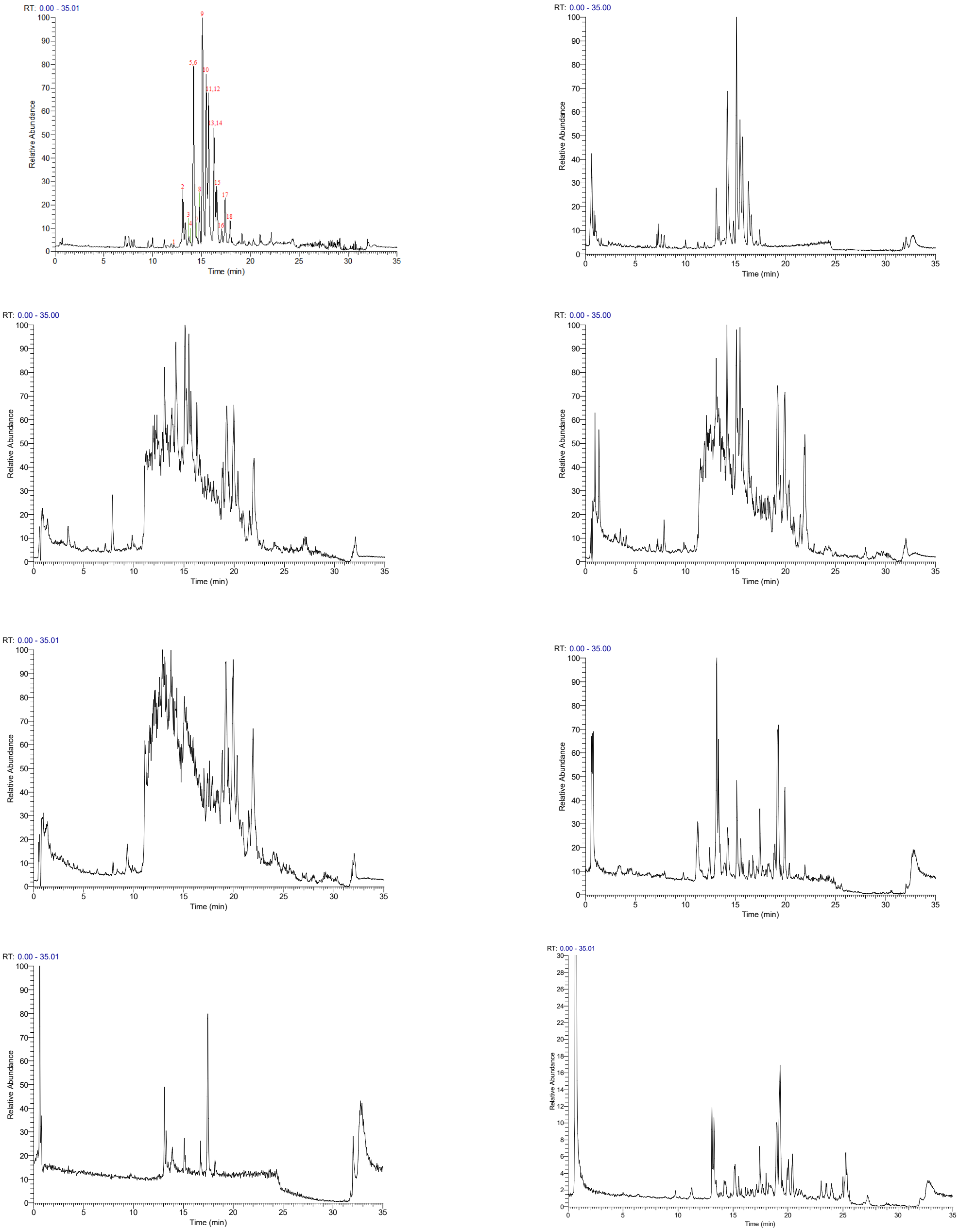

Supplementary Figure S1. Total ion chromatogram (TIC). The results of PMFs from UPLC-Q Exactive-Orbitrap HRMS in positive ion mode. (A) PMFs extract; (B) SGJ; (C)MB from intestinal wall metabolism group; (D) MB from intestinal flora metabolism group; (E) FVB; (F) AA; (G) CSF; (H) BT.

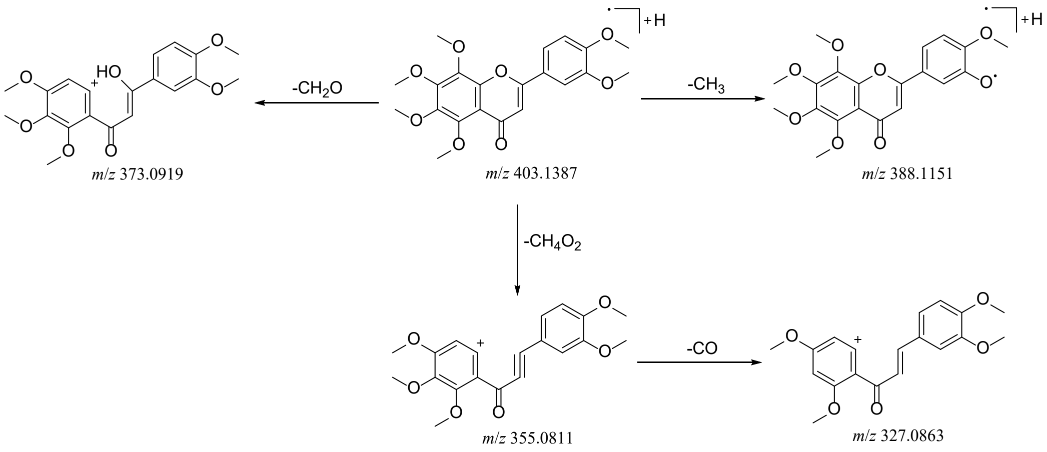

Supplementary Figure S2. Fragmentation pathways of NOB in positive ion mode.

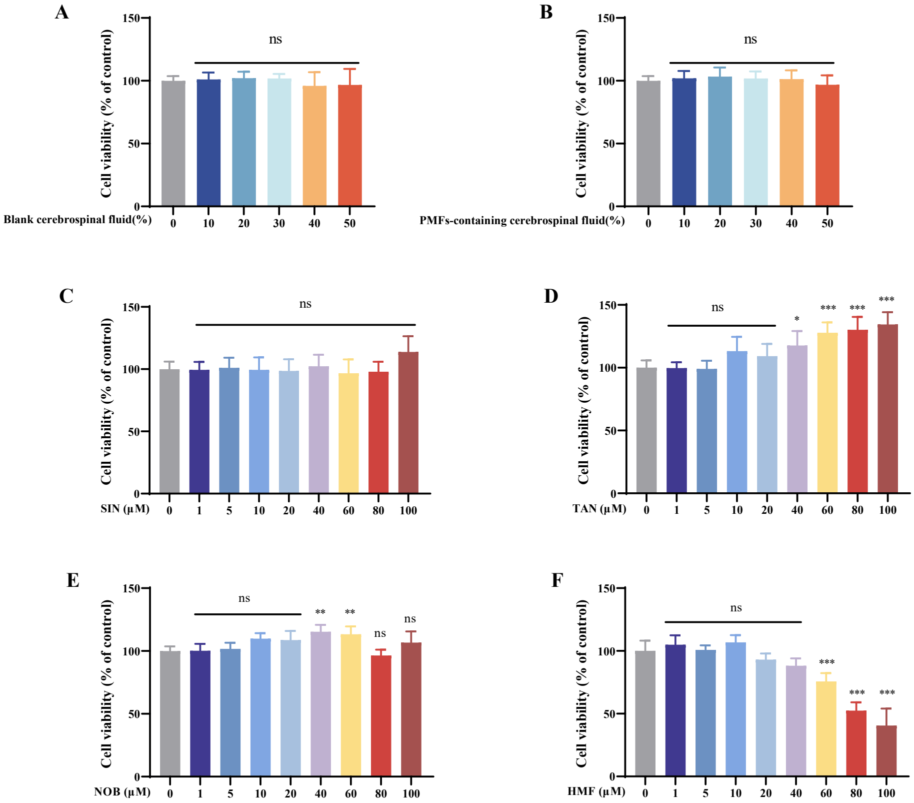

Supplementary Figure S3. Cell viability test. Effects of blank cerebrospinal fluid (A), PMFs-containing cerebrospinal fluid (B), SIN (C), TAN (D), NOB (E), and HMF (F) on the viability of BV2 microglial cells. ns represents no significant difference. *$p<0.05$, **$p<0.01$, ***p<0.00l, compared with the control group.

Supplementary Table S1. Summary of pharmacokinetic and pharmacodynamic properties of PMFs

PMFs	Pharmacokinetics				Pharmacodynamics	
	Biological matrices	Administeation	Parameters		Disease	Effects
Nobiletin	Plasma	Oral administration (5 mg/kg) [61]	$AUC_{(0-t)}$ (ng/mL*h):<br>$AUC_{(0-\infty)}$ (ng/mL*h):<br>$T_{1/2}$(h):<br>T_{max} (h):<br>CL (L/h/kg):<br>V (L/kg):<br>C_{max} (ng/mL):<br>Bioavailability (%):	2077 ± 141.6<br>2079.3 ± 141.9<br>2.4 ± 1.6<br>1<br>2.4 ± 0.2<br>8.2 ± 5.6<br>1254.6 ± 167.6<br>63.9%	Inflammatory bowel disease<br><br>Rheumatoid Arthritis	Anti-inflammatory: iNOS and COX-2↓[63]<br><br>Anti-inflammatory: IL-1β, MCP-1, IL-6, and TNF-α↓[64]
	Plasma	Intravenous administration (1 mg/kg) [61]	$AUC_{(0-t)}$ (ng/mL*h):<br>$AUC_{(0-\infty)}$ (ng/mL*h):<br>$T_{1/2}$(h):<br>CL (L/h/kg):<br>V (L/kg):<br>C_{max} (ng/mL):	650.3 ± 35.4<br>653.7 ± 31.7<br>4.1 ± 3.4<br>1.5 ± 0.1<br>9.3 ± 8.2<br>555.2 ± 101.1	Atherosclerotic	Platelet-derived growth factor, angiotensin II, and platelet aggregation ↓[65]
	Plasma	Oral administration (50 mg/kg) [62]	$AUC_{(0-t)}$ (ng/mL*h):<br>$AUC_{(0-\infty)}$ (ng/mL*h):<br>$T_{1/2}$(h):<br>T_{max} (h):<br>C_{max} (ng/mL):	7490<br>7840<br>1.8<br>1<br>1780	Cancer	iNOS↓, inducing antioxidative enzymes and arresting cell cycle progression [66]
	Brain	Oral administration (50 mg/kg) [62]	$AUC_{(0-t)}$ (ng/mL*h):<br>$AUC_{(0-\infty)}$ (ng/mL*h):<br>$T_{1/2}$(h):<br>T_{max} (h):<br>C_{max} (ng/mL):	20660<br>23200<br>11.42<br>1<br>4200	AD	Aβ↓: APP and BACE1 ↓[67]<br>Anti-inflammatory: IL-1β、TNF- α and IL-18 ↓[68]
Tangeretin	Plasma	Oral administration (5 mg/kg)[61]	$AUC_{(0-t)}$ (ng/mL*h):<br>$AUC_{(0-\infty)}$ (ng/mL*h):<br>$T_{1/2}$(h):<br>T_{max} (h):<br>CL (L/h/kg):<br>V (L/kg):<br>C_{max} (ng/mL):<br>Bioavailability (%):	167.7 ± 15.1<br>174.6 ± 19.7<br>5.3 ± 1.8<br>2<br>28.9 ± 3.3<br>214.5 ± 56.3<br>44.7 ± 2.5<br>46.10%	Diabetic	Anti-inflammatory, anti-oxidative, and modulating lipid homeostasis [71]
	Plasma	Oral administration (50 mg/kg) [69]	$AUC_{(0-t)}$ (ng/mL*h):<br>$T_{1/2}$(h):<br>T_{max} (h):<br>C_{max} (ng/mL):<br>Bioavailability (%):	3563 ± 1343.83<br>5.71 ± 1.19<br>5.67 ± 0.82<br>870 ± 330<br>27.11%	Cisplatin induced renal injury	Anti-infammatory cascade and anti-oxidative perturbations [72]

PMFs	Pharmacokinetics				Pharmacodynamics	
	Biological matrices	Administeation	Parameters		Disease	Effects
**Tangeretin**	Plasma	Intravenous administration (1 mg/kg)[61]	$AUC_{(0-t)}$ (ng/mL*h):<br>$AUC_{(0-\infty)}$ (ng/mL*h):<br>$T_{1/2}$(h):<br>CL (L/h/kg):<br>V (L/kg):<br>C_{max} (ng/mL):	72.7 ± 15.2<br>78.6 ± 15.9<br>5.5 ± 2.9<br>13.1 ± 2.4<br>101.9 ± 54.1<br>69.7 ± 22.2	AD	Inhibit BACE1 protein activity [73]
	Plasma	Intravenous administration (10 mg/kg)[70]	$T_{1/2}$(h):<br>CL (L/h/kg):<br>V (L/kg):<br>C_{max} (ng/mL):	2.77 ± 0.7<br>5.65 ± 1.21<br>4.20 ± 0.82<br>2470 ± 557	Cancer	Inducing apoptosis of human gastric cancer AGS cells [74]
**Sinensetin**	Plasma	Oral administration (*Orthosiphon stamineus Benth*. extract 10g/kg) [75]	$AUC_{(0-t)}$ (ng/mL*h):<br>$AUC_{(0-\infty)}$ (ng/mL*h):<br>$T_{1/2}$(h):<br>T_{max} (h):<br>C_{max} (ng/mL):	1.66 ± 1.16<br>1.76 ± 1.24<br>0.59 ± 0.23<br>0.38 ± 0.21<br>2.05 ± 1.90	Virus-triggered inflammation	Anti-inflammatory: IL-6, TNF-α, IL-8, and MCP-1↓ [78]
	Plasma	Oral administration (Bufei Jianpi granule extract 1.18 g/kg) [76]	$AUC_{(0-t)}$ (ng/mL*h):<br>$T_{1/2}$(h):<br>T_{max} (h):<br>C_{max} (ng/mL):	215.430 ± 4.683<br>7.893 ± 0.866<br>2.833<br>21.916 ± 1.947	Osteoarthritis	Anti-inflammatory: PGE2, IL-6, and TNF α↓ [79]
	Plasma	Oral administration (*Citri Reticulatae Pericarpium* extract 4 g/kg)[77]	$AUC_{(0-t)}$ (ng/mL*h):<br>$T_{1/2}$(h):<br>T_{max} (h):<br>C_{max} (ng/mL):	901.9 ± 424.0<br>2.9 ± 0.1<br>1.9 ± 1.2<br>171.4 ± 62.3	AD	Inhibit BACE1 protein activity [73]
**5,7,3',4'-tetramethoxyflavone**	Plasma	Oral administration (50 mg/kg)[80]	AUC_{last} (ng/mL*h):<br>T_{max} (h):<br>C_{max} (ng/mL):	2104.90 ± 580.31<br>4.00 ± 0.00<br>434.30 ± 156.96	Osteoarthritis	Anti-inflammatory: IL-1β, TNF-α, and PGE2↓ [81]
**3,5,6,7,8,3′,4′-heptamethoxyfavone**	-	-	-	-	Global cerebral ischemia	Rescued against ischemia-induced memory; Protected neuronal cell death in the CA1 cell layer; In creased the production of BDNF in the hippocampus [85]
**Isosinensetin**	Plasma	Oral administration (*Citri Reticulatae Pericarpium* extract 4 g/kg)[77]	$AUC_{(0-t)}$ (ng/mL*h):<br>$T_{1/2}$(h):<br>T_{max} (h):<br>C_{max} (ng/mL):	1085.5 ± 353.3<br>3.2 ± 0.3<br>4.3 ± 1.9<br>171.7 ± 47.3	-	-
	Plasma	Oral administration of 10 mg/kg[82]	$AUC_{(0-\infty)}$ (g/L*h):<br>$T_{1/2}$(h):<br>T_{max} (h):<br>C_{max} (μg/L):	806.5± 204.96<br>1.76 ± 0.23<br>0.5<br>367.49 ± 100.25		

PMFs	Pharmacokinetics				Pharmacodynamics	
	Biological matrices	Administeation	Parameters		Disease	Effects
5,7,3′,4′,5′-pentamethoxyflavone	Plasma	Oral administration 300 mg/kg[83]	AUC (nmol/mL):<br>C_{max} (nmol/mL):<br>$T_{1/2}$(min):<br>T_{max} (min):	1924<br>4.4<br>201<br>60	-	-
	Mucosa	Oral administration 300 mg/kg[83]	AUC (nmol/mL):<br>C_{max} (nmol/mL):<br>$T_{1/2}$(min):	520×10^3<br>3.425<br>140		
3,5,7,3′,4′-pentamethoxyflavone	Liver<br>Kidney<br>Lung<br>Testes<br>Brain<br>Plasma	Oral administration of 250 mg/kg *Kaempferia parviflora* extract [84]	AUC (h*μg/mL):<br>C_{max} (μg/mL):<br>$T_{1/2}$(h):<br>T_{max} (h):	3.65 ± 0.63<br>0.55 ± 0.05<br>3.12 ± 1.34<br>1..71 ± 0.36	-	-
3,5,7,4'-tetramethoxyflavone	Plasma	Oral administration (50 mg/kg)[80]	AUC_{last} (ng/mL*h):<br>$T_{1/2}$(h):<br>T_{max} (h):<br>CL (L/h/kg):<br>V (L/kg):<br>C_{max} (ng/mL):	4083.17 ± 1246.62<br>0.89 ± 0.55<br>2.17 ± 0.37<br>13.55 ± 4.68<br>17.75 ±12.20<br>1215.58 ± 232.25	-	-
5,6,7,4'-tetramethoxylflavone	Plasma	Oral administration (50 mg/kg)[80]	AUC_{last} (ng/mL*h):<br>$T_{1/2}$(h):<br>T_{max} (h):<br>CL (L/h/kg):<br>V (L/kg):<br>C_{max} (ng/mL):	101.35 ± 57.22<br>-<br>1.42 ± 0.61<br>-<br>-<br>55.60 ± 27.14	-	-
3,7,3',4'-tetramethoxyflavone	Plasma	Oral administration (50 mg/kg)[80]	AUC_{last} (ng/mL*h):<br>$T_{1/2}$(h):<br>T_{max} (h):<br>CL (L/h/kg):<br>V (L/kg):<br>C_{max} (ng/mL):	101.35 ± 57.22<br>-<br>1.42 ± 0.61<br>-<br>-<br>55.60 ± 27.14	-	-
3,5,7,2',4'-pentamethoxyflavone	Plasma	Oral administration (50 mg/kg)[80]	AUC_{last} (ng/mL*h):<br>$T_{1/2}$(h):<br>T_{max} (h):<br>CL (L/h/kg):<br>V (L/kg):<br>C_{max} (ng/mL):	2490.31 ± 492.05<br>0.53 ± 0.15<br>1.17 ± 0.37<br>20.76 ± 4.42<br>16.24 ± 7.17<br>1043.71 ± 252.45	-	-
3,5,7,3',4-pentamethoxyflavone	Plasma	Oral administration (50 mg/kg)[80]	AUC_{last} (ng/mL*h):<br>$T_{1/2}$(h):<br>T_{max} (h):<br>CL (L/h/kg):<br>V (L/kg):<br>C_{max} (ng/mL):	3212.44 ± 1362.71<br>0.46 ± 0.11<br>1.80 ± 0.40<br>18.82 ± 8.15<br>12.09 + 4.95<br>1397.64 ± 557.35	-	-

PMFs	Pharmacokinetics				Pharmacodynamics	
	Biological matrices	Administeation	Parameters		Disease	Effects
3,5,7,3',4',5'-hexamethoxylflavone	Plasma	Oral administration (50 mg/kg)[80]	AUC_{last} (ng/mL*h):	1880.36 ± 544.48	-	-
			$T_{1/2}$(h):	2.27 ± 0.59		
			T_{max} (h):	3.00 ± 0.00		
			CL (L/h/kg):	27.82 ± 8.44		
			V (L/kg):	94.78 + 51.79		
			C_{max} (ng/mL):	329.06 ± 75.626		
